# Pollination Across the Diel Cycle: A Global Meta‐Analysis

**DOI:** 10.1111/ele.70036

**Published:** 2024-12-31

**Authors:** Liam Kendall, Charlie C. Nicholson

**Affiliations:** ^1^ Centre for Environmental and Climate Science Lund University Lund Sweden; ^2^ Department of Biology Lund University Lund Sweden; ^3^ Department of Biology, Marine Biology, and Environmental Science Roger Williams University Bristol Rhode Island USA

**Keywords:** diurnal, floral traits, fruit set, nocturnal, pollination, pollination syndromes, seed set

## Abstract

The daily transition between day and night, known as the diel cycle, is characterised by significant shifts in environmental conditions and biological activity, both of which can affect crucial ecosystem functions like pollination. Despite over six decades of research into whether pollination varies between day and night, consensus remains elusive. We compiled the evidence of diel pollination from 135 studies with pollinator exclusion experiments involving 139 angiosperms. We used phylogenetic multi‐level meta‐analysis to test the influence of environmental conditions and plant functional traits on diel pollination differences. Our synthesis revealed an overall lack of difference in pollination between day and night; many plant species (90% of studied spp.) exhibit similar pollination success across the diel cycle. Diel pollination differences were partially explained by elevation: nocturnal pollination success was greater at low elevations, whereas diurnal pollination was more beneficial at higher elevations. Furthermore, floral traits related to pollinator attraction (odour, colour) and anthesis time influenced diel pollination differences. In the light of increasing anthropogenic pressures on biodiversity, as well as unique challenges for nocturnal biota, this synthesis underscores the diel complementarity of pollinators for many flowering plants and the importance of considering both nocturnal and diurnal pollinators in agricultural and conservation contexts.

## Graphical abstract

The daily transition between day and night, known as the diel cycle, is characterised by significant shifts in environmental conditions and biological activity, both of which can affect crucial ecosystem functions like pollination. Yet, despite over six decades of research into whether plant reproductive success varies between day and night, consensus remains elusive. Our synthesis revealed an overall lack of difference in pollination success between day and night, suggesting many plant species (90% of studied spp.) exhibit similar pollination success across the diel cycle.

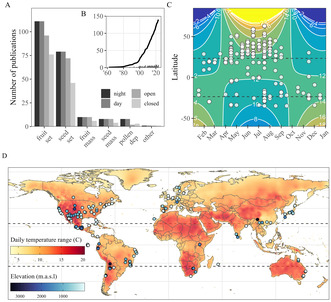

## 1 Background

Few environmental fluctuations are more consistent than the transition of day to night. The 24‐h (diel) cycle can see considerable variation in resources (e.g., sunlight), abiotic conditions (e.g., temperature), and biotic interactions (e.g., predation). Species have evolved suites of traits to exploit daily environmental fluctuations, leading to diel differences in ecosystem function. In a review of five key ecosystem functions, Cox and Gaston (2024) concluded that our understanding of the distinct but linked processes of nocturnal and diurnal ecosystems is underdeveloped. A persistent diurnal bias in ecological research (Park 1940; Gaston 2019) is worth addressing for several reasons. First, nocturnality is widespread, with 30% of vertebrates and more than 60% of invertebrates exhibiting nocturnal activity (Hölker et al. 2010), with insect activity on average 31.4% higher at night than in the day (Wong and Didham 2024). Likewise, approximately one‐third of angiosperm families contain species that benefit from nocturnal pollination (Borges, Somanathan, and Kelber 2016). Second, diel‐differentials exist for many anthropogenic pressures (Gaston, Gardner, and Cox 2023), for example, light or noise pollution (McMahon, Rohr, and Bernal 2017) or diel asymmetry in warming—minimum temperatures (often night occurring) are increasing at much greater rates than are maximum temperatures (Donat and Alexander 2012; Cox et al. 2020). It remains true that nocturnal ecology must be considered before our knowledge of a community, its functions, and its responses to global change can be considered complete (Park, Lockett, and Myers 1931).

Pollination is a crucial ecosystem function for crops and wildflowers (Klein et al. 2007; Ollerton, Winfree, and Tarrant 2011). The relative contribution to plant reproductive success varies extensively amongst pollinator groups (Herrera 1987; Wilson and Thomson 1991; Page et al. 2021), and is the product of (1) a plant's reliance on animal vectors for successful reproduction (i.e., pollination dependency (Eckert et al. 2010)), and (2) a pollinator's visitation rate and per‐visit pollen transfer efficiency (i.e., pollination effectiveness (Stebbins 1970)). Both nocturnal and diurnal flowering plants differ considerably in their pollination dependency and nocturnal and diurnal pollinators can differ considerably in their pollination effectiveness. Yet, a strong diurnal bias persists in pollination research (Macgregor and Scott‐Brown 2020; Buxton et al. 2022), which limits our understanding and appreciation of diel variation in pollination outcomes.

There are well‐documented examples of pollination by nocturnal lepidoptera (Brantjes 1978; Anderson, Rotheray, and Mathews 2023), beetles (Consiglio and Bourne 2001; Grant et al. 2021), and mammals (Goldingay, Carthew, and Whelan 1991; Fleming et al. 2001), and many plant species are visited by a range of diurnal and nocturnal pollinators (Fleming and Holland 1998; Knop et al. 2018; Siqueira et al. 2018; Alison et al. 2022; Fijen et al. 2023). The contribution to reproductive success by nocturnal pollinators can be minimal for some plant species (e.g., Morse and Fritz 1983), such as for plants with low pollinator dependency, yet for other plants, nocturnal pollinators are the most effective and/or only pollen vector (e.g., Young 2002). Furthermore, disregarding nocturnal pollinators can lead to misleading inferences of plant species pollination requirements (Alison et al. 2022).

Associations between floral traits (e.g., scent, colour, floral morphology, heat generation) and nocturnal or diurnal pollinator functional groups (Baker 1961; Fenster et al. 2004; Faegri and Pijl 2013) may help explain diel pollination differences. These ‘pollination syndromes’ have precedent, as research has shown that specialising pollination on a diel functional group can improve plant reproductive outcomes, for example, greater pollen transfer from nocturnal moths with large foraging ranges (Kawakita and Kato 2004). Yet, the fallibility of syndromes is well known (Ollerton et al. 2009, but see Dellinger 2020). For example, plants conforming to the moth‐pollination syndrome (i.e., white, fragrant, tubular flowers) are, in fact, often pollinated by a diversity of nocturnal and diurnal insects (Slauson 2000; Funamoto and Ohashi 2017). Moreover, floral receptivity over a 24‐h cycle can increase visitation and provide resilience against local pollinator extinctions (Walton et al. 2020; Shibata and Kudo 2023). Nonetheless, it remains widely assumed that specific floral traits are associated with nocturnal or diurnal pollination (Valdivia and Niemeyer 2006).

Abiotic conditions may also influence diel pollination differences. Natural nocturnal light levels can be several orders of magnitude lower than daytime levels (Borges, Somanathan, and Kelber 2016). These low‐light conditions can affect plant signalling and pollinator attraction. Moreover, total daylight hours will vary with latitude and time of year, and research suggests the relative contribution from nocturnal pollinators could decrease with latitude (Sletvold et al. 2012; Chapurlat, Agren, and Sletvold 2015), where seasonal periods of pollinator activity coincide with increased daylength. In temperate and dry regions, night temperatures tend to be lower, with minimum daily temperatures commonly occurring at night. Pollination rates are also known to vary altitudinally (Arroyo, Armesto, and Primack 1985; Adedoja, Kehinde, and Samways 2018), potentially due to harsher environmental conditions at higher elevations (Cruden 1972; Dellinger et al. 2023). Nocturnal, diurnal, or crepuscular activity may be an adaptation to avoid these unfavourable conditions (Herrera 1992; Heinrich 1993; Willmer and Stone 1997). Alternatively, the arid hypothesis for nocturnal flowering and pollination (Borges, Somanathan, and Kelber 2016) posits that plants in dry conditions should preferentially bloom at night since it reduces the water demands of flowering (e.g., Galen, Sherry, and Carroll 1999; Galen 2000). This is also reflected in evidence that nocturnal pollination is found in 67.8% of the 31 families with CAM photosynthesis (Borges, Somanathan, and Kelber 2016). This hypothesis has mixed support, for example, within Cactaceae where bee and bird pollination is more common than bat and moth pollination (Gorostiague, Ollerton, and Ortega‐Baes 2023). Although night‐time abiotic conditions will differ between latitude and climate zone, environmental diel variation may nonetheless create unique temporal niches to which plants and pollinators have adapted.

Numerous studies have directly tested the relative contribution of nocturnal and diurnal pollination through exclusion experiments, whereby plants or flowers are bagged to prevent either diurnal or nocturnal pollinator visitation. These treatment groups (night and day pollination) are often paired with additional pollination treatments, including control flowers (open pollination) or supplemental pollen addition (hand pollination). Although previous reviews of nocturnal pollination exist (Baker 1961; Borges, Somanathan, and Kelber 2016; Macgregor and Scott‐Brown 2020; Buxton et al. 2022), none synthesise evidence of the relative contribution of nocturnal and diurnal pollinators to plant reproductive success. Given these common experimental designs, we undertook a meta‐analysis to investigate diel pollination differences, and the biotic or abiotic factors that may explain these differences. We define pollination benefit for a diel period as the plant reproductive success if pollinators visiting outside of that period are experimentally excluded. We ask the following questions:

1. To what extent does pollination benefit differ between day and night amongst flowering plants?
2. Are diel pollination differences explained by (i) environmental conditions (i.e., daylength, daily temperature range, and elevation), and (ii) plant functional traits?

## 2 Methods

### 2.1 Literature Search and Inclusion

We conducted our literature search in October 2023 using the following piloted search string: (“nocturnal*” OR “night*”) and (“pollin*”) and (“success*” OR “pollen*” OR “fruit*” OR “seed*” OR “effic*” OR “effective*” OR “visit*”). This search string identified 1893 papers in the Web of Science (WoS) from 9 databases, including WoS Core Collection, CABI, BIOSIS Previews, ProQuest, SciELO, and Zoological Record. In addition, we collated references from two recent reviews of nocturnal pollination (Macgregor and Scott‐Brown 2020; Buxton et al. 2022), which yielded an additional 306 references. After removing duplicates, we used Rayyan (Ouzzani et al. 2016) to screen all 1950 unique bibliographic records. Through screening abstracts, we identified 275 papers as possible candidates for inclusion. After screening the full text of these papers, we identified 136 that contained potential data for meta‐analysis. We included studies if they 1) conducted a pollinator exclusion experiment in which pollination was completely inhibited during both day and night; 2) measured pollination benefit as either fruit set, fruit mass, seed set, seed mass, or pollen deposition; 3) reported sample sizes and descriptive statistics (e.g., means, boxplots). Our first criterion excluded papers that used taxon‐specific exclusion methods, (e.g., wire cages against nocturnal mammals Kleizen, Midgley, and Johnson 2008). Our second criteria excluded most measures of male fitness (e.g., pollen removal). A PRISMA inclusion chart (Figure A1.1) is provided in Appendix S1.

### 2.2 Data Extraction

In addition to experimental exclusion of day and night pollination, we recorded responses to treatments where all pollinator visitation was excluded (i.e., autogamy, complete exclusion, or bagged treatments) and where no exclusion occurred (i.e., control or open pollination treatments). We extracted the sample size, mean, and variance, including from figures when these values were not reported in text or tables using WebPlotDigitizer (Rohatgi 2020). All variance measurements were converted to standard deviation. We did not record the identity of pollinator taxa observed in studies as there was large variation in how this information was reported (see Discussion).

### 2.3 Missing Data Imputation

When results were reported as boxplots, we used the function *metaDigitise* (Pick, Nakagawa, and Noble 2019) to extract and estimate means and variance. Fruit set results were often reported without variance. As such, where fruit set was reported as the number of fruit resulting from a number of flowers, we calculated the theoretical variance assuming a binomial distribution. We imputed missing variances for 29 comparisons from studies that did not report standard deviation but for which we could extract sample sizes. Within each pollination outcome measure, we modelled standard deviation as a function of the sample size, mean pollination outcome, and treatment (day pollination, night pollination, or open pollination), following Bishop and Nakagawa (2021). For seed set, we included interactions amongst the predictors (*F*_19,507_: 69.26, *R* ^2^ = 0.71), whereas for seed mass (*F*_5,23_: 23.31, *R* ^2^ = 0.80) and pollen deposition (*F*_5,23_: 25.04, *R* ^2^ = 0.81), we included only additive terms given the small sample sizes. There was one instance where the study year was not reported, and authors did not respond to email inquiry. For this missing year, we used the study's publication date and imputed a study year based on the average time between study date and publication date for all other articles (4.9 years).

### 2.4 Effect Size Calculation

We calculated effect sizes as the standardised mean difference (SMD), otherwise known as Hedges' *g* (Hedges 1981). We chose to use SMD over other effect sizes because it can handle zeroes and includes a correction for small sample sizes (Nakagawa and Santos 2012), which occurred with our data. For each pollination benefit measure, we calculated SMD for three treatment comparisons: (1) between night and day pollination, (2) between day and open pollination, and (3) between night and open pollination. For the first comparison, effect sizes > 0 indicate night pollination was more effective than day pollination, whilst for the other comparisons, effect sizes < 0 mean that day or night pollination was less effective than open pollination. Our inclusion criteria ensured all studies contained a day vs. night comparison (*n* = 136); however, open pollination was reported in 83.8% of studies (*n* = 114) and so the sample size for these treatment comparisons differs. In addition, 64.7% of studies (*n* = 88) reported both open and closed pollination, with which we calculated plant species pollination dependency as the SMD between these treatments. We calculated effect sizes in R (R Core Development Team 2021) using the escalc function in the *metafor* package (v. 2.1–0, Viechtbauer 2010).

### 2.5 Moderators

We collected information on several moderator variables to explain heterogeneity in the data or to account for phylogenetic similarity. We directly extracted five variables from the included studies: (1) study location—if latitude and longitude were not reported, then coordinates were estimated based on place names or maps; (2) study year—for studies with field seasons that overlapped two years, we used the first year; (3) study duration—start and end months of exclusion experiments; (4) plant crop status, and (5) exposure time—length, in hours, that pollinators were excluded during each diel period. Where exposure time was not explicitly stated, we imputed missing values using extracted data on daylength for study localities (see below).

To explore the influence of biogeography, we obtained each study's day length, daily temperature range, and elevation. We use the CBM model (Forsythe et al. 1995) to calculate day length according to solar declination, a study's latitude, and day of year based on the median date of the experiment's start and end months. Using a study's location, median date, and year, we extracted the month‐long average of daily temperature ranges based on the Climatic Research Unit Time Series (CRU TS, v.4.07) dataset (Harris et al. 2020). Elevation was obtained for each study location by extracting point elevations from the Amazon Web Services Terrain Tiles using the *elevatr* package (v.0.99.0, Hollister and Shah 2017).

Plant phenotype may influence diel pollination effectiveness. Following Lanuza et al. (2023), we collected information on the following pollination‐relevant plant traits (Table S1): plant lifespan, life form, photosynthetic pathway, breeding system, flower colour, flower symmetry, flower shape, anthesis time, nectar presence, odour presence, flower width (mm), flower length (mm), style length (mm), and plant height (m). The included studies reported many of these trait values, and we filled in missing values by searching the literature and referencing botanical keys. Where quantitative traits (e.g., flower width) could not be found we accessed herbarium specimens hosted on GBIF and measured dimensions of at least five flowers. When sex differences existed between quantitative traits (e.g., for dioecious plants), we took measurements from female flowers. We excluded one plant species (*Mitrastemon yamamotoi*, Mitrastemonaceae) prior to analyses, as it lacked photosynthesis, and could not be reliably imputed to either the CAM or C_4_ pathway (thus, *N* = 135 studies). We also re‐classified the only two plant species with capitulum flowers (family Asteraceae) as open flowers, as well as one plant species with blue flowers (*Adenophora jasionifolia*) and one with brown flowers (*Cullenia exarillata*) as purple and yellow, respectively. Furthermore, where anthesis time was unobtainable (*n* = 24 plant species), we classified these as having variable (“both”) anthesis. Lastly, as pollination dependency may structure diel pollination differences, we included this as a moderator for the subset of studies and plant species for which we had these values (see above).

We assessed correlations between continuous traits and environmental variables using Pearson's correlation coefficient. We further calculated measures of associations between pairs of nominal traits using Cramer's V, and between continuous variables and nominal traits with the *R* ^2^ from linear regressions (Figure A1.2). Flower width, length and style length were found to be highly correlated. Thus, we elected to model only style length, as a measure of functional flower size related to pollination. In contrast, daylength, DTR and elevation were uncorrelated, and thus retained in our analyses.

Plants with traits associated with night or day pollination might have shared evolutionary history. After resolving species names to current nomenclature using the *worldflora* package (v.1.14–1, Kindt 2020), we constructed a phylogeny with the *V.PhyloMakeR2* package (v.0.1.0; Jin and Qian 2019) by pruning a dated plant phylogeny (Smith and Brown 2018), and added unrepresented species using the default scenario (i.e., missing genera and species were placed at the basal node of the family or genus respectively). This time‐calibrated phylogeny allowed us to construct a phylogenetic covariance matrix. We use these phylogenies and covariance matrices in our meta‐regression models (see below).

### 2.6 Multi‐Level Meta‐Analysis and Regression

**FIGURE 1:**
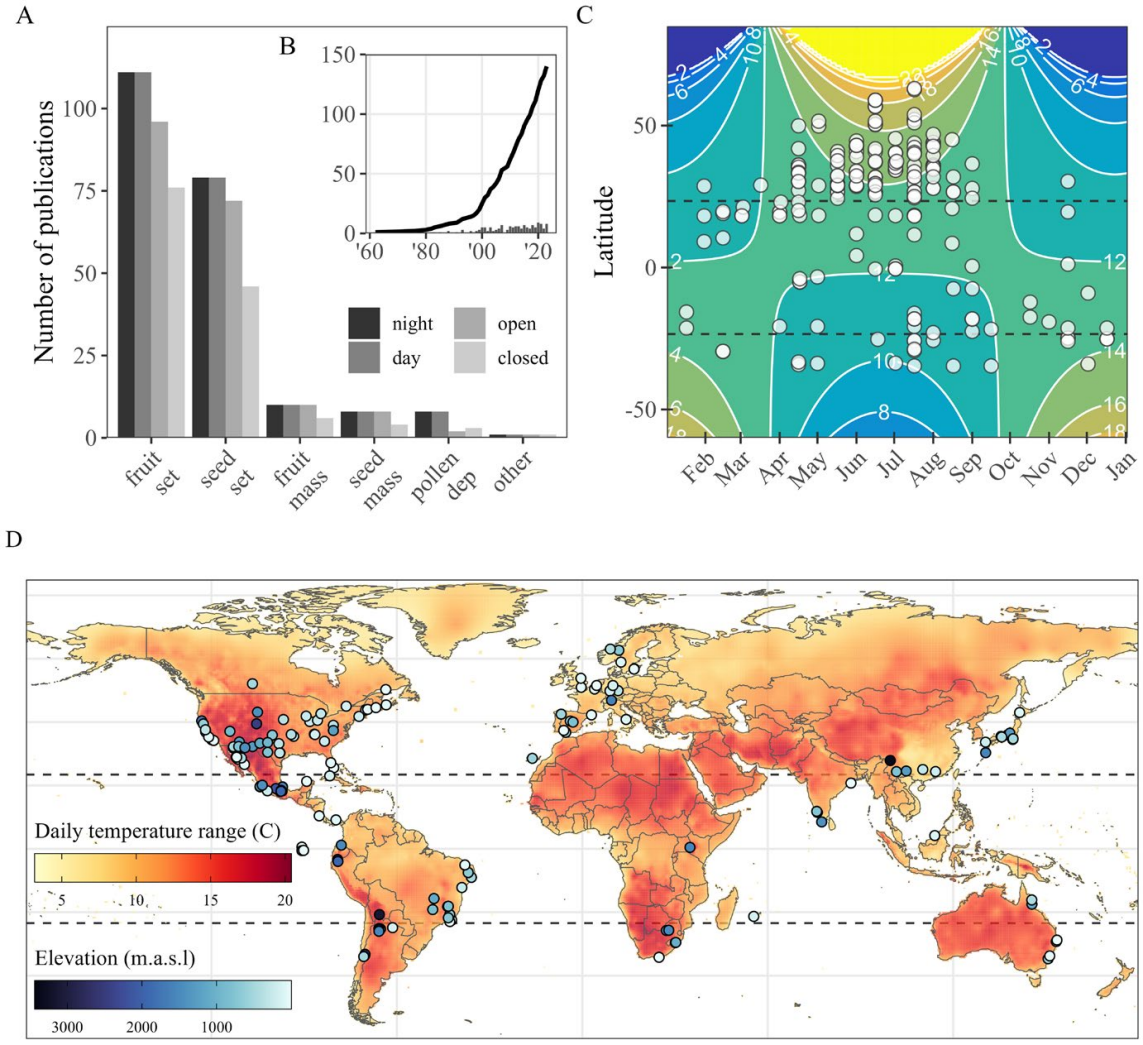
The research into diel pollination differences examined different pollination outcomes between night, day, open, and closed pollination treatments (A), progressed over time (B), occurred across a range of daylengths (C), temperature conditions, and elevations (D). The time series (B) shows the cumulative (line) and annual (bars) number of publications. Each study's daylength (C, hours) was computed using each study's location and median date. Daily temperature range and elevation (D) were extracted based on study location (see Methods).

Meta‐analysis and meta‐regression were undertaken using the rma.mv function within the ‘*metafor’* package (v.4.4–0, Viechtbauer 2010). Prior to analyses, we removed five effect sizes (day vs. night: *n* = 3, day vs. open: *n* = 2, open vs. closed: *n* = 1), as these were extreme values (i.e., SMD > 10 or < −10). These outliers came from three studies (Valiente‐Banuet et al. 1997; Barthelmess, Richards, and McCauley 2006; Anderson et al. 2015) and were the result of extremely large differences in reproduction success between treatment groups (e.g., > 1300 seeds (night) vs. 0 seeds (day), Valiente‐Banuet et al. 1997). This yielded 1273 effect sizes (day vs. night: 400, day vs. open: 342, night vs. open: 352, open vs. closed: 179), which were distributed across different pollination benefit metrics and treatment comparisons (Figure 1A). Likewise, this resulted in a different number of analysed plant species for each treatment comparison (day vs. night: *n* = 138, day vs. open: *n* = 112, night vs. open = 112, open vs. closed = 94). We first fitted multi‐level meta‐analysis models to assess overall trends for each diel pollination difference (i.e., SMD between day vs. night pollination, day vs. open pollination, and night vs. open pollination), as well as pollination dependency (closed vs. open pollination). We found that diel pollination differences were explained by pollination exposure time (Figure A1.3; day vs. night: *z* = −2.522, *p* = 0.012), but not always (day vs. open: *z* = 1.726, *p* = 0.084; night vs. open: *z* = 1.342, *p* = 0.18). To account for this bias, we included a diel exposure quotient as a fixed effect in all models, expressed as the ratio between day and night exposure hours (day: night pollination), or between either diel period and a 24‐h period for open pollination comparisons. Given the hierarchical structure of our dataset (i.e., including both multiple effect sizes resulting from the same study, different pollination measurements, as well as species with shared evolutionary history), we included five random effects, (i) study, to account for multiple effect sizes resulting from the same study, (ii) pollination benefit metric type, (iii & iv) phylogenetic and non‐phylogenetic species effects, and (v) an observation‐level effect for residual heterogeneity amongst effect sizes. We estimated total heterogeneity in each diel pollination comparison and that associated with each random effect using *I* ^*2*^ (Higgins and Thompson 2002). Additionally, we assessed the phylogenetic signal for each diel pollination difference, as Pagel's λ (Pagel 1999), using marginal species‐level predictions from our meta‐analytic models with the *phytools* package (v.2.1–1, Revell 2012).

We then used multi‐level meta‐regression to assess if diel pollination differences were structured by (i) diel pollination benefit metric, (ii) plant species traits and pollination dependency, and (iii) environment. To assess differences amongst diel pollination benefit metrics, we fitted models with pollination benefit metric as a fixed effect, along with a reduced random effect structure (study, phylogenetic and non‐phylogenetic species effects, and observation‐level effect).

We then modelled diel pollination difference for all diel pollination comparisons in relation to individual traits and environmental variables, including diel exposure quotient and with the same random structure as our meta‐analytical models. We assessed variables individually as the strong dependencies amongst multiple variables limited inference based upon multi‐predictor models. We compared linear and quadratic terms for continuous variables (e.g., elevation, dependency) to test for non‐linear relationships. We then evaluated model heterogeneity using Cochrane's *Q* test and goodness of fit with the marginal *R* ^2^ (Nakagawa and Schielzeth 2013). *p*‐values from Cochrane's *Q* tests were adjusted for multiple stesting using the Benjamini‐Yekutieli method (Benjamini and Yekutieli 2001). Model estimates were considered statistically significant if their 95% confidence intervals did not cross zero. We visualised our results using modified code from the *orchaRd* package (v2.0, Nakagawa et al. 2023).

### 2.7 Publication Bias, Limitations, and Sensitivity Analysis

We tested for publication bias using three approaches. First, we plotted SMD against its standard error (square‐root of sampling variance), to look for asymmetry in funnel plots. Second, we used a modified version of the Egger's regression test (Egger et al. 1997) for multi‐level meta‐analytic models, which regresses the SMD on its standard error, whilst accounting for the random effect structure. Here, if the model intercept is significantly different from zero, this is indicative of publication bias or “small study effects”. Third, we tested for time‐lag effects (Jennions and Møller 2002) by modelling SMD as a function of publication year, together with the random effect structure described above.

## 3 Results

### 3.1 Attributes of Diel Pollination Studies

Our final dataset consisted of a total of 1094 effect sizes, from 135 studies, and 139 plant species (85 genera and 37 families), resulting in 400 comparisons between day vs. night pollination, 342 between day pollination and open pollination, and 352 between night and open pollination. Most studies reported pollination outcomes as fruit set (56%, 609 effect sizes), followed by seed set (35%, 388 effect sizes), and fruit mass (*n* = 48), seed mass (*n* = 30), and pollen deposition (*n* = 19) comprised a minority of data (9%; Figure 1A). Pollination experiments were conducted between 1962 and 2022 (Figure 1B), typically during summer months and with an average daylength of 13 h ± 1.56 (mean ± SD), daily temperature range of 13°C ± 3.67, and elevation of 678 m ± 749 (Figure 1C,D). Research was conducted on every continent, with 43% of studies from North America (*n* = 58), 21% from South America (*n* = 28), 15% from Europe (*n* = 20), 13% from Asia (*n* = 18), and 4% from each of Africa (*n* = 6) and Oceania (*n* = 5) (Figure 1D). The top three most represented plant families in terms of number of species were Cactaceae (*n* = 23), Caryophyllaceae (*n* = 11), and Asparagaceae (*n* = 11). Amongst the plant species that compared closed and open pollination (68%, *n* = 94 species, *n* = 179 effect sizes), we found strong evidence for pollination dependency (*z* = 3.919, *p* < 0.001, Figure A1.4).

### 3.2 Day vs. Night Pollination

**FIGURE 2:**
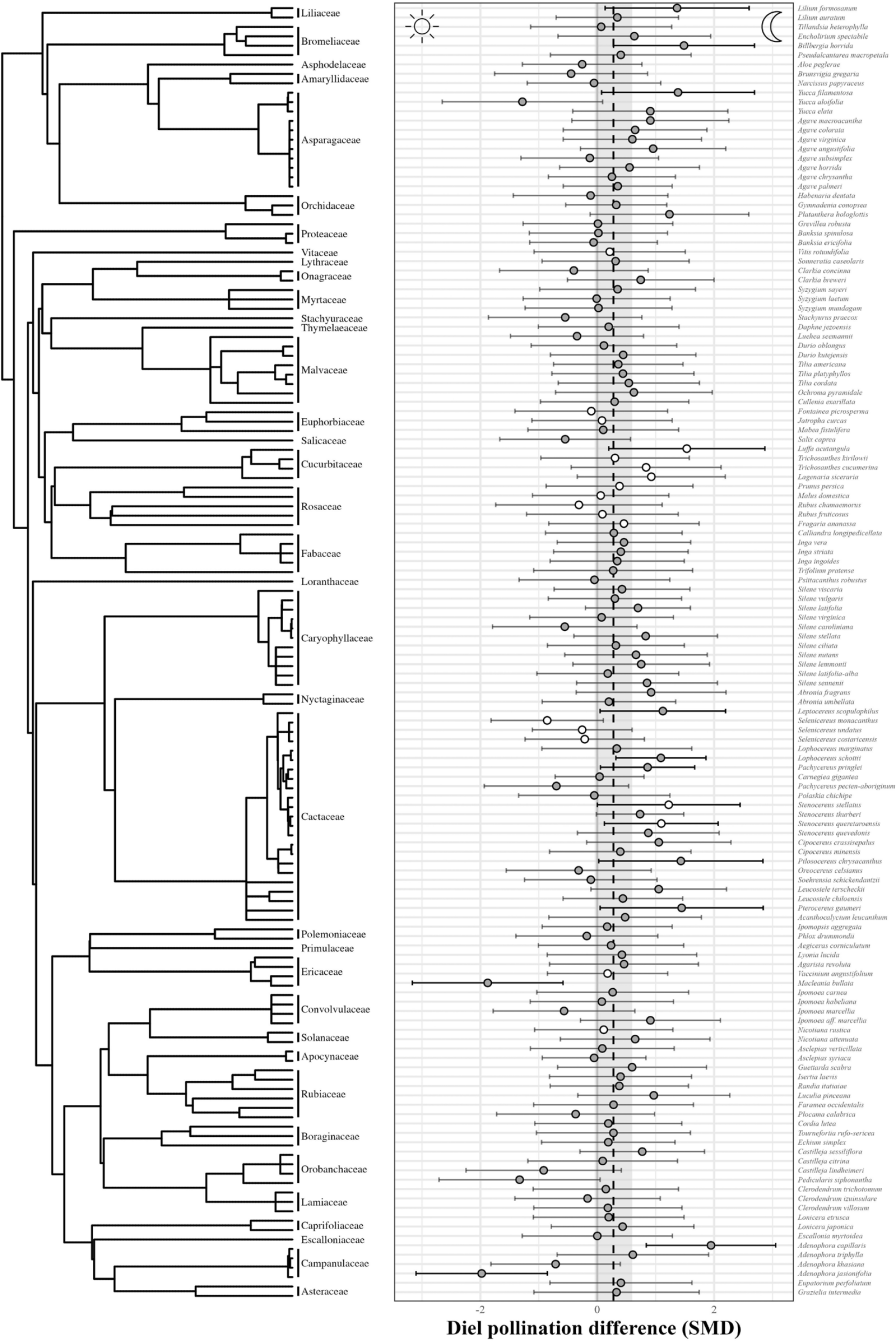
Phylogeny of plant species included in meta‐analysis (left), and species‐level predictions of diel pollination difference between day and night pollination (right). Primary dots and error bars indicate marginal species‐level mean estimates ±95% confidence intervals. Filled circles indicate wild plant species, and open circles are crop species. Vertical dashed line and shaded rectangle indicate meta‐analytical mean estimate and 95% confidence interval.

Phylogenetic meta‐analysis revealed that there were no overall significant differences between day and night pollination (Figure 2), with 14 species (10%) having SMD values that do not overlap zero. Total heterogeneity was high (*I* ^2^ = 98%), with the most being attributed to the non‐phylogenetic species effect (44%), followed by individual effects (26%), study (24%), pollination benefit metric (2%) and species phylogeny (2%). We found no evidence of phylogenetic signal in differences between day and night pollination (*λ* = 0.014, *p* = 0.741). We found that day or night pollination were generally less effective than a full 24‐h (open) pollination period but henceforth focus on day vs. night pollination and report the full results for open pollination vs. day or night pollination in Appendix S2.

**FIGURE 3:**
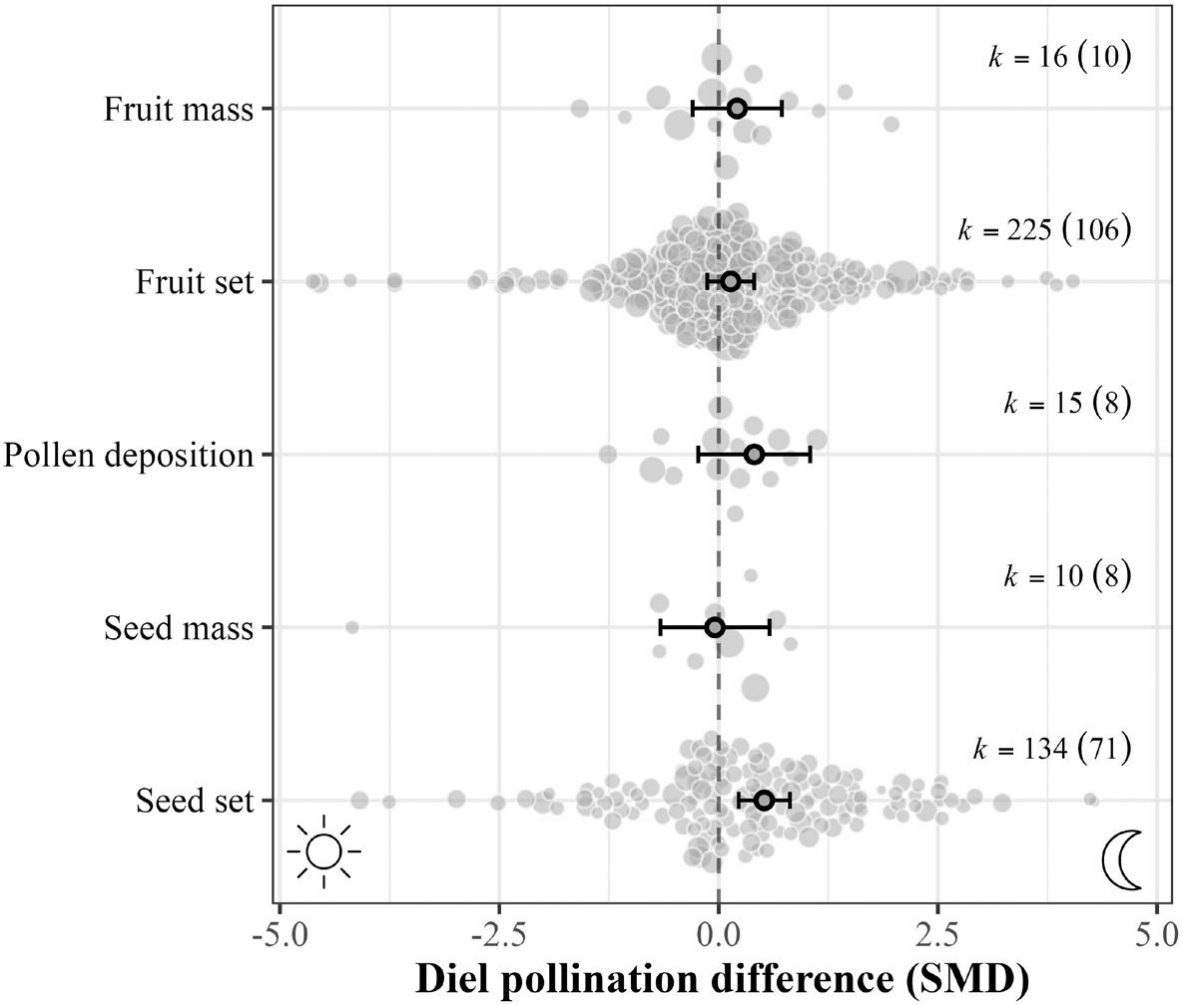
Diel pollination differences (Standardised mean differences, SMD) between day and night pollination for each pollination outcome measure. Primary dots and error bars indicate marginal mean estimates and 95% confidence intervals. Background points indicate individual effect sizes, in which size is proportional to the inverse of the standard error of the effect size. *k =* the number of effect sizes along with the number of studies in parentheses.

At the pollination metric level (Figure 3), seed set resulting from night pollination was significantly greater than from day pollination (0.521 [0.229, 0.812]), whereas the diel pollination difference of all other outcomes (fruit mass, fruit set, pollen deposition and seed mass) did not differ from zero.

### 3.3 Diel Pollination Differences in Relation to Environmental Variables and Plant Traits

**FIGURE 4:**
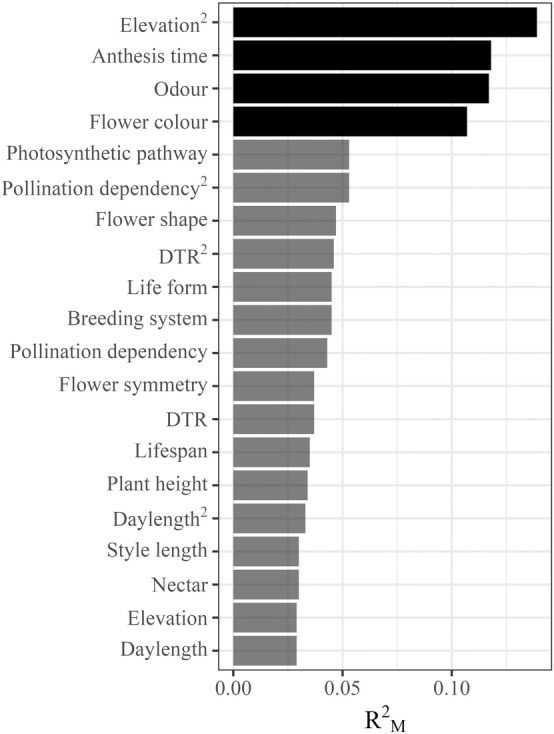
Coefficient of determination (marginal *R* ^2^) for each environmental and trait variable in relation to diel pollination differences between day and night pollination. Solid columns are those variables for which Cochrane's *Q* test was significant (adj. *p* < 0.05), whereas transparent columns were non‐significant (adj. *p* > 0.05).

Elevation, as well as three plant functional traits explained a significant amount of variation in diel pollination differences (Table S2, Figure 4).

**FIGURE 5:**
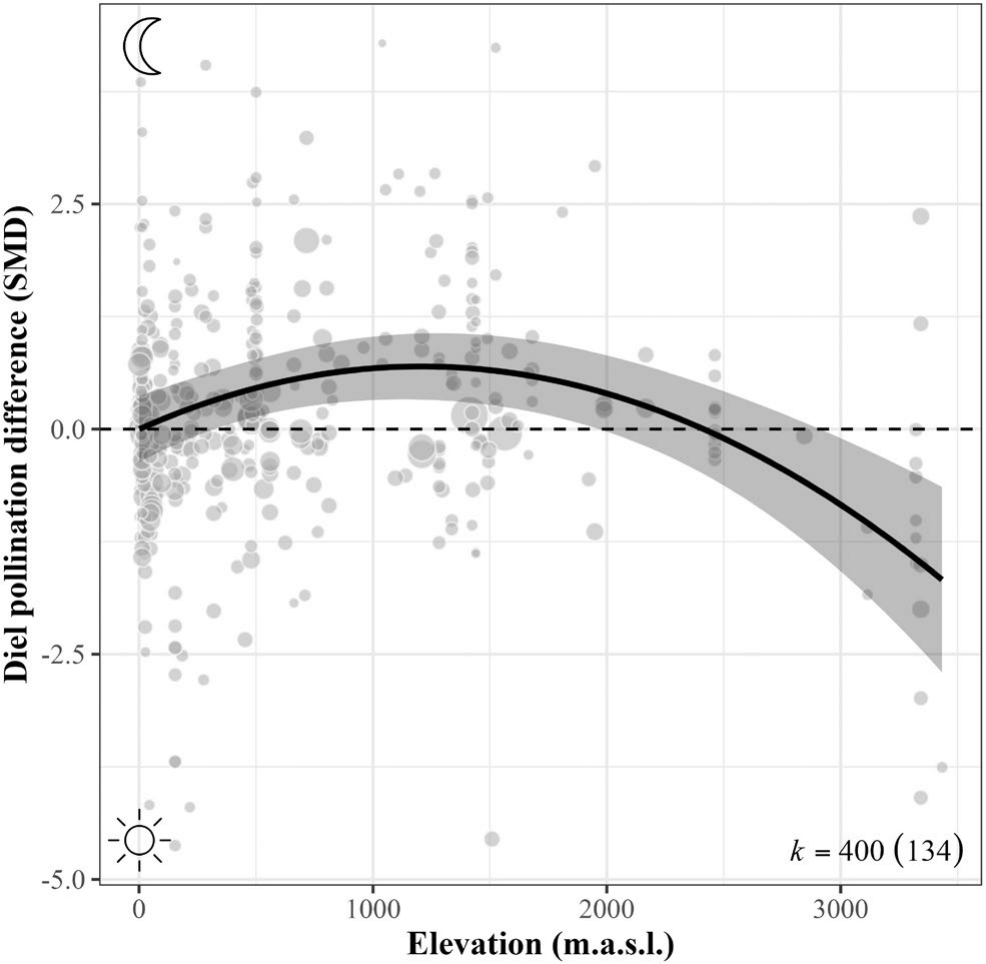
Diel pollination differences (Standardised mean difference, SMD) between day and night pollination in relation to elevation (m.a.s.l.). Solid line and shaded ribbon indicate predicted line of best fit and 95% confidence intervals. Background points indicate individual effect sizes, in which size is proportional to the inverse of the standard error of the effect size. *k =* the number of effect sizes along with the number of studies in parentheses.

We found that the success of nocturnal pollination relative to day pollination was highest at mid elevations, before declining at higher elevations (linear term: *z* = 3.184, *p* = 0.002, quadratic term: *z* = −4.815, *p* < 0.001, Figure 5).

**FIGURE 6:**
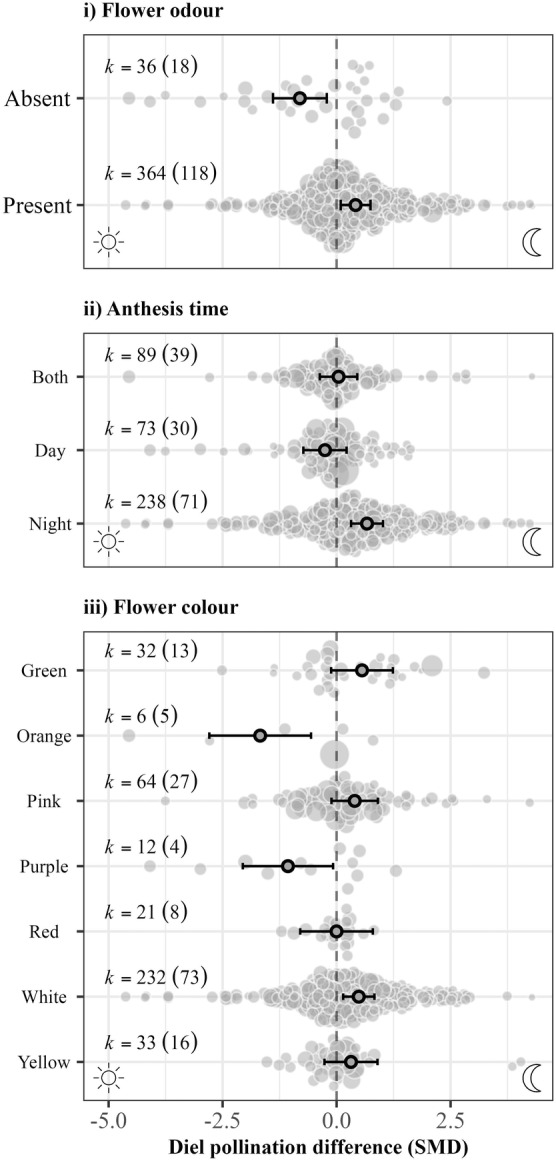
Diel pollination differences (Standardised mean difference) in relation to plant traits: (i) flower odour, (ii) anthesis time, and (iii) flower colour. Primary dots and error bars indicate marginal mean estimates and 95% confidence intervals. Background points indicate individual effect sizes, in which size is proportional to the inverse of the standard error of the effect size. *k =* the number of effect sizes along with the number of studies in parentheses.

Colour, odour, and anthesis time influenced diel pollination differences (Figure 6). Diurnal pollination success was higher, relative to nocturnal pollination, amongst species with flowers lacking discernible odour (−0.805 [−1.394, −0.215]), whereas the presence of odour led to the reverse (0.417 [0.091, 0.743]). In addition, nocturnally blooming plant species had significantly higher pollination success from nocturnal pollination relative to diurnal pollination (0.668 [0.317, 1.018]). Plants with orange and purple flowers exhibited greater pollination success from diurnal pollination relative to nocturnal pollination (orange: −1.675 [−2.791, −0.56]; purple: −1.066 [−2.057, −0.076]), whereas white flowers benefited more so from nocturnal pollination (0.487 [0.145, 0.829]).

### 3.4 Publication Bias and Sensitivity Analysis

The data showed little evidence of publication bias in terms of funnel plot asymmetry (Figure A1.5). Results from the Egger's tests suggested there was no evidence for asymmetry (*z* = −1.011, *p* = 0.312). Additionally, we found no evidence for a time‐lag effect (year effect: *z* = 0.563, *p* = 0.574).

## 4 Discussion

Through the most quantitative synthesis of pollination across the diel cycle to date, we demonstrate that many plant species (90% of studied spp.) exhibit similar pollination success across the diel cycle. Although adaptation to conditions in one part of the diel cycle may confer fitness disadvantages in other periods, this was only the case for a subset of studied species (see Figure 2). Elevation and plant life history traits explained variation in diel pollination differences, but not plant phylogeny, daylength, or daily temperature range. Daytime and nighttime activity play an important role in temporal niche partitioning and ecosystem function. Given that comparable rates of nocturnal and diurnal pollination success is potentially widespread, important directions for future research include (i) understanding the contribution and management of nocturnal crop pollinators (Buxton et al. 2022) and (ii) understanding the magnitude and effect of anthropogenic pressures on pollination in the night‐time environment, for example, night‐time warming or light pollution (Tougeron and Sanders 2023).

Pollination success did not differ between day and night pollination, except for greater seed set from nocturnal pollination. For the plant species included in our analysis, authors hypothesised greater nocturnal pollination success was caused by, for example, increased dispersal of pollen (Young 2002; Barthelmess, Richards, and McCauley 2006), more efficient pollen receipt or deposition (Miyake and Yahara 1998; Anderson, Rotheray, and Mathews 2023), and/or the timing of anthesis or stigma receptivity (Groman and Pellmyr 1999; Young and Gravitz 2002). Is the contribution of nocturnal pollinators to seed set detected here representative of angiosperm pollination more broadly? It's unlikely because many studies chose plants for which there was an expectation of nocturnal pollination. Given this selection bias, perhaps the more surprising result is the general absence of diel pollination differences. These results, together with our findings that day or night pollination is often less effective than a full 24‐h pollination period (Appendix S2), point towards cathermality (activity during both daytime and night‐time) of plant reproductive strategies and pollination complementarity over time periods. Diel complementarity, wherein both diurnal and nocturnal pollinators contribute to plant reproductive success (Jennersten and Morse 1991; Devoto, Bailey, and Memmott 2011; Amorim, Galetto, and Sazima 2013; Aguilar‐Rodriguez et al. 2016; Funamoto and Sugiura 2021), provides functional redundancy across the diel cycle, and may provide resilience against pressures that disproportionately act during any one period (e.g., heat during the day, artificial light at night). Yet, substantial variation in the degree of diel pollination difference existed, and we found some support that this is explained by environmental variables and plant traits. We discuss these in turn.

Diel variation in pollination success was structured along an elevational gradient, such that nocturnal pollination was more beneficial amongst plant species at low—to—mid elevations (500–1500 m.a.s.l.), whereas diurnal pollination was more effective at higher elevations (> 2750 m.a.s.l.). The decline of nocturnal pollination success with altitude may be attributed to the reduced activity of night‐active pollinators. For example, studies in the two cactus species *Oreocerus celsianus*, (Larrea‐Alcazar and Lopez 2011) and *Echinopsis schnickendantzi* (Alonso‐Pedano and Ortega‐Baes 2012) at > 3000 m demonstrated that diurnal pollination by hummingbirds or bees respectively, surpassed nocturnal pollination, owing to the infrequent presence of nocturnal pollinators. Interestingly, whilst *O. celsianus* exhibits floral traits suited for hummingbird pollination, those of *E. schnickendantzi* are suggestive of a moth‐pollination syndrome (e.g., presence of odour, white flowers), indicating the importance of flexibility of pollination syndromes for plant reproduction in harsh environments. Furthermore, previous studies have demonstrated elevational turnover in (sub‐)tropical pollination systems, most notably from ectothermic invertebrates to endothermic vertebrates at high altitudes (Cruden 1972; Dellinger et al. 2023). As the environmental conditions at high altitudes associated with diurnal foraging activity are arguably less severe than night‐time conditions, our results suggest that altitudinal shifts in pollination systems can also result in diel turnover in pollination success across both vertebrate and invertebrate taxa.

The underlying causes of improved nocturnal pollination success at lower elevations remain ambiguous yet may reflect underlying elevational patterns in biodiversity. Both species and interaction diversity in plant‐pollinator networks can peak at mid elevations (Hoiss, Krauss, and Steffan‐Dewenter 2015), and diel network comparisons highlight that nocturnal pollination networks can be more species rich than diurnal networks (Walton et al. 2020), in line with general patterns in insect activity across the diel cycle (Wong and Didham 2024). Thus, given that pollinator diversity can be linked with greater pollination success (Dainese et al. 2019), observed trends may indicate previously unappreciated diversity of nocturnal insects within a large proportion of studied pollination systems.

Our results support classical notions of pollination syndromes based on plant traits. Nocturnal pollination success was higher, relative to diurnal pollination, for white, odour‐producing flowers with night anthesis. Conversely, plants with scentless and orange or purple flowers with day anthesis benefitted more from diurnal pollination. Although pollinators use both visual and olfactory cues (Riffell and Alarcón 2013), odour can be a more reliable and longer ranging cue at night. Our results support the importance of odour as a critical stimulus for nocturnal pollinators. We hesitate to conclude on the adaptive significance of flower colour, given (1) small sample sizes (e.g., six effect sizes for orange flowers), (2) a prevalence of non‐significant differences across all colours, and (3) flower pigment is often genetically correlated (e.g., through pleiotropy) with other traits that could be under selection (Mckinnon and Pierotti 2010; Wessinger and Rausher 2012).

We found a strong association between nocturnal pollination and increased pollination success in flowers that open at night. This association is suggestive of an adaptive response due to greater pollinator efficiency amongst nocturnally active pollinators compared to their diurnal counterparts. For instance, Young (2002) proposed that nyctinasty, where flowers open at night and close during the day, led to increased pollination success in *Silene alba*, as moths transported pollen across greater distances, leading to improved outcrossing rates, whilst simultaneously limiting pollination by less effective diurnal pollinators. In addition, synchronisation between flower anthesis timing and stigma receptivity has also been proposed as a potential mechanism for diel variation in pollination outcomes (Young and Gravitz 2002). However, Young and Gravitz found no evidence of this, and our results also do not support this notion as we found no corresponding pattern between diurnal pollination and daytime‐blooming flowers. Furthermore, several studies indicate that the timing of pollination throughout the day has minimal impact on pollination outcomes, as demonstrated through timed hand pollination experiments (Haber and Franke 1982; Kwak and Jennersten 1986; Wolff, Braun, and Liede 2003; Martins et al. 2020). As such, our results contend that nocturnal anthesis likely represents an adaptive strategy, alongside other traits which affect pollinator preference such as odour and colour (Matsumoto et al. 2015) due to the improved effectiveness of nocturnal pollinators for these plant species.

Our meta‐analysis reveals blind spots in our understanding of pollination across the diel cycle. Given the importance of pollination for crop production, we were surprised that most studies (87%) focused on non‐cultivated plants. Interestingly, a number of studies found no difference between day and night pollination for crop species expected to be primarily bee, which is to say, day‐pollinated (Cutler et al. 2012; Robertson et al. 2021; Fijen et al. 2023). This highlights the potential importance of nocturnal pollination and risk of yield gaps if nocturnal pollinators are not part of crop pollination management plans. However, diurnal and nocturnal pollinators may not respond equivalently to agricultural management. For example, planting herbaceous wildflowers to support pollinators may be beneficial for bees (Albrecht et al. 2020), yet moth communities are likely to benefit from increasing tree and shrub density (Bates et al. 2014; Ellis and Wilkinson 2021). One direction for future crop pollination research is to investigate the degree of nocturnal pollination dependency and what management practices can co‐benefit diurnal and nocturnal pollinator communities.

Our meta‐analysis has limitations. First, lunar cycles can regulate the activity of many insects (Warrant and Dacke 2010; Kronfeld‐Schor et al. 2013), including pollinators (Kerfoot 1967; Young et al. 2021), but studies rarely reported lunar phase or date information at resolutions fine enough to examine this. Future experiments could consider lunar phase as an important covariate in the design and analysis of diel pollination research. Second, we focus on biotic pollination. Environmental factors that differ between day and night, such as air turbidity and humidity would likely affect wind pollination (Timerman and Barrett 2021). Last, we do not report the pollinator taxa responsible for nocturnal or diurnal pollination. Studies used a variety of methods (e.g., point counts, pan traps), sampled over a range of time periods (e.g., minutes to hours), and reported pollinator identity at different taxonomic resolutions. This lack of standardisation is understandable. Conducting nocturnal pollinator observations is demanding and logistically challenging (Wong and Didham 2024), particularly when experiments require in‐person observation. Additionally, we found that imbalanced pollination exclusion time influenced diel pollination differences. Technological innovations in biodiversity monitoring, such as eDNA, acoustic recording devices, and automated imaging, could overcome these challenges, provide more standardised monitoring, and adjust the diurnal bias in ecological research.

## 5 Conclusions

Diel variation in conditions, resources, and interactions can form the basis of temporal niches in which species have evolved traits to maximise fitness (Kronfeld‐Schor and Dayan 2003). Temporal partitioning across a diel cycle may facilitate plant coexistence, for example through a reduction in competition for pollinators (e.g., Stone, Willmer, and Nee 1997), but our results do not support widespread specialisation to specific diel periods. Rather, for many plants, reproductive success is flexible to the timing of pollination. Where diel pollination differences do exist, they are explained by flower colour, odour, and anthesis time—plant traits well known to be related to pollinator attraction. Nonetheless, our data show that exceptions to syndrome‐based expectations are numerous, and thus we discourage assuming pollinator activity period based on plant traits alone. We did not find support for large‐scale biogeographical patterns of diel pollination difference, for example across latitudinal gradients of daylength (Munguia‐Rosas et al. 2009; Sletvold et al. 2012) or temperature range (Borges, Somanathan, and Kelber 2016). Our meta‐analysis addresses the ‘lack of strong experimental evidence’ of diel pollination differences (Buxton et al. 2022) and highlights where there is more to learn about the drivers, consequences, and responses to diurnal and nocturnal pollination. Diel variation in ecosystem functioning is a frontier of ecological research (Cox and Gaston 2024), and anthropogenic pressures on the night‐time environment are increasing (Gaston, Gardner, and Cox 2023). Redressing a diurnal bias in ecological research will continue to yield novel insights and the evidence needed to ensure ecosystem functioning in daytime and night‐time environments.

## Author Contributions

L.K.K., and C.C.N. contributed equally to study conception, design and methodology, data acquisition, data analysis, data interpretation, and writing.

## Conflicts of Interest

The authors declare no conflicts of interest.

## Supporting information


Appendix S1.

## Data Availability

Data and code for reproducing statistical results and generating figures are available via Figshare (https://doi.org/10.6084/m9.figshare.26004538.v1) and through an open GitHub repository (https://github.com/liamkendall/bumble‐bat).
