# Supplementary material for: Pollination Across the Diel Cycle: A Global Meta‐Analysis
Source: Ecol Lett. 2024 Dec 31;28(1):e70036. doi: 10.1111/ele.70036 (PMC11687351; doi:10.1111/ele.70036)
Supplement: Supplementary file 1 — Appendix S1. [file ELE-28-0-s001.docx]

**APPENDIX 1**

**Supporting information**

**Table S1.** Trait values for plant species reported in included studies. Qualitative traits were scored based on flower symmetry (actinomorphic: A; zygomorphic: Z), life span (perennial: P; short-lived: S), life form, photosynthetic (PS) pathway, flower shape, breeding system (hermaphrodite: h, diecious: d; monoecious: m), anthesis time (day-blooming: D, night-blooming: N, both or unclear: B), the presence or absence (p vs. a) of nectar and odour, and colour (green: G; orange: O; pink: Pi; purple: Pu; red: R; white: W; yellow: Y). For quantitative traits, we converted all floral measurements to mm and all plant heights to m. Where values were reported as a range (e.g., 6-10 m) we used the midpoint. We include both the species name reported in study articles and the species name used to construct phylogenies based on (Smith and Brown 2018).

| **Reported species** | **Current species** | **Plant family** | **Flower symmetry** | **lifespan** | **Life form** | **PS pathway** | **Flower shape** | **Breeding system** | **Anthesis time** | **nectar** | **odour** | **colour** | **Flower width (mm)** | **Flower length (mm)** | **Style length (mm)** | **Plant height (m)** |
| --- | --- | --- | --- | --- | --- | --- | --- | --- | --- | --- | --- | --- | --- | --- | --- | --- |
| Abronia fragrans | Abronia fragrans | Nyctaginaceae | A | P | herb | C3 | tube | h | N | p | p | Pi | 8 | 25 | 15 | 0.7 |
| Abronia umbellata | Abronia umbellata | Nyctaginaceae | A | S | herb | C3 | tube | h | B | p | p | Pu | 16 | 20 | 8 | 0.075 |
| Echinopsis leucantha | Acanthocalycium leucanthum | Cactaceae | A | P | herb | CAM | tube | h | N | p | a | W | 113.62 | 198.14 | 150 | 1.25 |
| Adenophora capillaris | Adenophora capillaris | Campanulaceae | A | P | herb | C3 | campanulate | h | N | p | p | W | 4.95 | 13.9 | 27.25 | 0.75 |
| Adenophora jasionifolia | Adenophora jasionifolia | Campanulaceae | A | P | herb | C3 | campanulate | h | D | p | a | Pu | 29.19 | 18.44 | 22.18 | 0.35 |
| Adenophora khasiana | Adenophora khasiana | Campanulaceae | A | P | herb | C3 | campanulate | h | N | p | a | Pu | 24.65 | 20.59 | 25.37 | 1 |
| Adenophora triphylla | Adenophora triphylla | Campanulaceae | A | P | herb | C3 | campanulate | h | N | p | a | Pu | 9.5 | 9.5 | 17 | 1.5 |
| Aegiceras corniculatum | Aegiceras corniculatum | Primulaceae | A | P | tree | C3 | brush | h | D | p | p | W | 15 | 12 | 10.35 | 6 |
| Agarista revoluta | Agarista revoluta | Ericaceae | A | P | tree | C3 | campanulate | h | B | p | p | W | 6.1 | 8.7 | 4.4 | 2.7 |
| Agave angustifolia | Agave angustifolia | Asparagaceae | A | P | herb | CAM | tube | h | B | p | p | W | 12.5 | 65 | 95 | 0.75 |
| Agave chrysantha | Agave chrysantha | Asparagaceae | A | P | herb | CAM | tube | h | D | p | p | Y | 12.5 | 40 | 57.5 | 1 |
| Agave colorata | Agave colorata | Asparagaceae | A | P | herb | CAM | tube | h | D | p | p | G | 12.5 | 47.5 | 65 | 2.5 |
| Agave horrida | Agave horrida | Asparagaceae | A | P | herb | CAM | tube | h | N | p | p | Y | 17.5 | 40 | 55 | 0.45 |
| Agave macroacantha | Agave macroacantha | Asparagaceae | A | P | herb | CAM | tube | h | N | p | p | G | 9 | 57.5 | 62.5 | 0.45 |
| Agave palmeri | Agave palmeri | Asparagaceae | A | P | herb | CAM | tube | h | D | p | p | G | 17.5 | 40 | 50 | 0.45 |
| Agave subsimplex | Agave subsimplex | Asparagaceae | A | P | herb | CAM | tube | h | B | p | p | Y | 15 | 37.5 | 52.5 | 0.275 |
| ManfRa virginica | Agave virginica | Asparagaceae | A | P | herb | CAM | tube | h | B | p | p | G | 4 | 20 | 32.5 | 1 |
| Aloe peglerae | Aloe peglerae | Asphodelaceae | A | S | herb | CAM | tube | h | N | p | a | Y | 13 | 30 | 55 | 0.4 |
| Asclepias syriaca | Asclepias syriaca | Apocynaceae | A | P | herb | C3 | open | h | B | p | p | Pi | 12.5 | 9 | 0.41 | 1.5 |
| Asclepias verticillata | Asclepias verticillata | Apocynaceae | A | P | herb | C3 | open | h | B | p | p | Pi | 4.2 | 3.33 | 0.375 | 0.45 |
| Banksia ericifolia | Banksia ericifolia | Proteaceae | A | P | shrub | C3 | brush | h | B | p | p | O | 57.5 | 150 | 25 | 6 |
| Billbergia horrida | Billbergia horrida | Bromeliaceae | A | P | herb | CAM | tube | h | N | p | p | G | 55 | 25 | 50 | 0.4 |
| Durio grandiflorus | Boschia grandiflora | Malvaceae | A | P | tree | C3 | open | h | D | a | p | W | 60 | 15 | 25 | 11.5 |
| Brunsvigia gregaria | Brunsvigia gregaria | Amaryllidaceae | A | P | herb | C3 | tube | h | B | p | a | R | 35 | 40 | 40 | 0.185 |
| Calliandra longipedicellata | Calliandra longipedicellata | Fabaceae | A | P | shrub | C3 | brush | h | N | p | p | R | 60 | 50 | 60 | 2 |
| Carnegiea gigantea | Carnegiea gigantea | Cactaceae | A | P | herb | CAM | tube | h | N | p | p | W | 24.9 | 112.2 | 53.9 | 8 |
| Castilleja citrina | Castilleja citrina | Orobanchaceae | Z | P | herb | C3 | tube | h | B | p | p | Y | 3.5 | 32.5 | 34.5 | 0.25 |
| Castilleja lindheimeri | Castilleja lindheimeri | Orobanchaceae | Z | P | herb | C3 | tube | h | B | p | p | O | 2.5 | 37.5 | 40.5 | 0.3 |
| Castilleja sessiliflora | Castilleja sessiliflora | Orobanchaceae | Z | P | herb | C3 | tube | h | B | p | p | W | 3 | 42.5 | 44.5 | 0.22 |
| Cipocereus crassisepalus | Cipocereus crassisepalus | Cactaceae | A | P | herb | CAM | tube | h | N | p | p | W | 55.9 | 55.7 | 84 | 3 |
| Cipocereus minensis | Cipocereus minensis | Cactaceae | A | P | herb | CAM | tube | h | N | p | p | W | 32.8 | 46.1 | 27.4 | 2 |
| Clerodendrum izuinsulare | Clerodendrum izuinsulare | Lamiaceae | A | P | tree | C3 | tube | h | B | p | p | W | 11.47 | 26.25 | 33.47 | 7.5 |
| Clerodendrum trichotomum | Clerodendrum trichotomum | Lamiaceae | A | P | tree | C3 | tube | h | D | p | p | W | 14.67 | 25.29 | 42.81 | 7.5 |
| Clerodendrum molle | Clerodendrum villosum | Lamiaceae | A | P | shrub | C3 | tube | h | B | p | p | W | 1.75 | 25 | 25 | 3.25 |
| Cordia lutea | Cordia lutea | Boraginaceae | A | P | shrub | C3 | tube | h | D | p | p | Y | 30 | 30 | 20 | 8 |
| Cullenia exarillata | Cullenia exarillata | Malvaceae | A | P | tree | C3 | tube | h | D | p | p | brown | 13.5 | 45 | 54 | 27.5 |
| Daphne jezoensis | Daphne jezoensis | Thymelaeaceae | A | P | shrub | C3 | tube | d | B | p | p | Y | 10.8 | 6.4 | 2.7 | 0.2 |
| Durio kutejensis | Durio kutejensis | Malvaceae | A | P | tree | C3 | open | h | N | p | p | R | 110 | 40 | 72.5 | 12 |
| Durio oblongus | Durio oblongus | Malvaceae | A | P | tree | C3 | open | h | D | p | p | W | 95 | 37.5 | 67.5 | 25 |
| Echium simplex | Echium simplex | Boraginaceae | A | P | herb | C3 | tube | h | D | p | p | W | 5 | 12.5 | 17.5 | 3 |
| Encholirium spectabile | Encholirium spectabile | Bromeliaceae | A | P | herb | CAM | tube | h | N | p | p | G | 20 | 15 | 10 | 2.5 |
| Escallonia myrtoidea | Escallonia myrtoidea | Escalloniaceae | A | P | tree | C3 | campanulate | h | B | p | p | W | 85 | 65 | 65 | 6 |
| Eupatorium perfoliatum | Eupatorium perfoliatum | Asteraceae | A | P | herb | C3 | capitulum | h | B | p | p | W | 3 | 5 | 6 | 1 |
| Faramea cyanea | Faramea occidentalis | Rubiaceae | A | P | tree | C3 | tube | h | N | p | p | W | 14 | 17 | 9 | 9 |
| Grazielia intermedia | Grazielia intermedia | Asteraceae | A | P | herb | C3 | capitulum | d | D | p | p | W | 2 | 4 | 6 | 2.5 |
| Grevillea robusta | Grevillea robusta | Proteaceae | Z | P | tree | C3 | brush | h | D | p | p | O | 2 | 23 | 17.5 | 17.5 |
| Guettarda scabra | Guettarda scabra | Rubiaceae | A | P | shrub | C3 | tube | h | N | p | p | W | 11.66 | 15.1 | 16.9 | 3.25 |
| Gymnadenia conopsea | Gymnadenia conopsea | Orchidaceae | Z | P | herb | C3 | papilionaceous | h | D | p | p | Pi | 10.34 | 15 | 1.5 | 0.237 |
| Inga ingoides | Inga ingoides | Fabaceae | A | P | tree | C3 | brush | h | D | p | p | W | 5 | 14 | 73 | 20 |
| Inga striata | Inga striata | Fabaceae | A | P | tree | C3 | brush | h | D | p | p | W | 4 | 10 | 71 | 20 |
| Inga vera | Inga vera | Fabaceae | A | P | tree | C3 | brush | h | D | p | p | W | 6 | 14 | 45 | 20 |
| Ipomoea carnea | Ipomoea carnea | Convolvulaceae | A | P | shrub | C3 | tube | h | B | p | p | Pi | 88.6 | 79.1 | 18.8 | 1.5 |
| Ipomoea habeliana | Ipomoea habeliana | Convolvulaceae | A | P | vine | C3 | tube | h | N | p | p | W | 60 | 110 | 150 | 8 |
| Ipomoea marcellia | Ipomoea marcellia | Convolvulaceae | A | P | vine | C3 | tube | h | D | p | p | W | 22.7 | 57.5 | 55.94 | 1.75 |
| Ipomopsis aggregata | Ipomopsis aggregata | Polemoniaceae | A | S | herb | C3 | tube | h | N | p | p | R | 2.5 | 32.5 | 25.5 | 0.9 |
| Isertia laevis | Isertia laevis | Rubiaceae | A | P | tree | C3 | tube | h | N | p | p | W | 32.4 | 41 | 42 | 15 |
| Jatropha curcas | Jatropha curcas | Euphorbiaceae | A | P | tree | CAM | campanulate | m | D | p | p | G | 8.47 | 4.44 | 7.59 | 12.5 |
| Lagenaria siceraria | Lagenaria siceraria | Cucurbitaceae | A | S | vine | C3 | tube | m | N | p | p | W | 95 | 9.4 | 3.5 | 9 |
| Leptocereus scopulophilus | Leptocereus scopulophilus | Cactaceae | A | P | herb | CAM | tube | h | N | p | p | W | 36 | 53 | 37 | 4 |
| Echinopsis chiloensis | Leucostele chiloensis | Cactaceae | A | P | herb | CAM | tube | h | N | p | p | W | 85 | 150 | 90.4 | 4.5 |
| Echinopsis terscheckii | Leucostele terscheckii | Cactaceae | A | P | herb | CAM | tube | h | N | p | a | W | 163.8 | 177.7 | 153.9 | 15 |
| Lilium auratum | Lilium auratum | Liliaceae | A | P | herb | C3 | open | h | D | p | p | W | 140 | 110 | 120 | 2.5 |
| Lilium formosanum | Lilium formosanum | Liliaceae | A | P | herb | C3 | open | h | B | p | p | W | 110 | 130 | 140.5 | 1.4 |
| Lonicera etrusca | Lonicera etrusca | Caprifoliaceae | Z | P | vine | C3 | tube | h | N | p | p | W | 2.5 | 32.9 | 42.7 | 4 |
| Marginatocereus marginatus | Lophocereus marginatus | Cactaceae | A | P | herb | CAM | tube | h | N | p | p | R | 15.7 | 34.7 | 22.88 | 8.5 |
| Lophocereus schottii | Lophocereus schottii | Cactaceae | A | P | herb | CAM | tube | h | N | a | p | Pi | 6.1 | 30 | 50 | 3 |
| Luculia pinceana | Luculia pinceana | Rubiaceae | A | P | shrub | C3 | tube | h | B | p | p | Pi | 30 | 27.5 | 33 | 6 |
| Luehea seemannii | Luehea seemannii | Malvaceae | A | P | tree | C3 | open | m | N | p | p | W | 15 | 7.5 | 10 | 22.5 |
| Luffa acutangula | Luffa acutangula | Cucurbitaceae | A | S | herb | C3 | tube | m | N | p | p | Y | 52.2 | 11 | 3.5 | 6 |
| Lyonia lucida | Lyonia lucida | Ericaceae | A | P | shrub | C3 | campanulate | h | B | p | p | Pi | 3.5 | 7 | 6 | 3.75 |
| Mabea fistulifera | Mabea fistulifera | Euphorbiaceae | A | P | tree | C3 | open | m | N | p | p | R | 13 | 26.6 | 26.6 | 6.5 |
| Macleania bullata | Macleania bullata | Ericaceae | A | P | shrub | C3 | tube | h | B | p | a | O | 5 | 42 | 43.8 | 3 |
| Malus domestica | Malus domestica | Rosaceae | A | P | tree | C3 | open | h | B | p | p | W | 35 | 15 | 12.5 | 3.5 |
| Mitrastemon yamamotoi | Mitrastemon yamamotoi | Mitrastemonaceae | A | P | herb | NA | open | h | B | p | p | W | 20 | 15 | 25 | 0.025 |
| Ipomoea aff. Marcellia | Ipomoea aff. Marcellia | Convolvulaceae | A | P | vine | C3 | tube | h | N | p | p | W | 18.42 | 22.73 | 42.33 | 1.75 |
| Narcissus papyraceus | Narcissus papyraceus | Amaryllidaceae | A | P | herb | C3 | tube | h | B | p | p | W | 30 | 14.5 | 16 | 0.375 |
| Nicotiana attenuata | Nicotiana attenuata | Solanaceae | A | S | herb | C3 | tube | h | N | p | p | W | 12.5 | 35 | 30 | 1.25 |
| Nicotiana rustica | Nicotiana rustica | Solanaceae | A | S | herb | C3 | tube | h | D | p | p | Y | 6.9 | 18 | 23 | 1.06135 |
| Ochroma pyramidale | Ochroma pyramidale | Malvaceae | A | P | tree | C3 | tube | h | N | p | p | W | 200 | 115 | 150 | 30 |
| Oreocereus celsianus | Oreocereus celsianus | Cactaceae | A | P | herb | CAM | tube | h | N | p | p | Pi | 60 | 78.1 | 75 | 6 |
| Pachycereus pecten-aboriginum | Pachycereus pecten-aboriginum | Cactaceae | A | P | herb | CAM | tube | h | N | p | p | W | 62 | 77 | 45 | 8 |
| Pachycereus pringlei | Pachycereus pringlei | Cactaceae | A | P | herb | CAM | tube | h | N | p | p | W | 24.2 | 102.2 | 53.2 | 7 |
| Pedicularis siphonantha | Pedicularis siphonantha | Orobanchaceae | Z | P | herb | C3 | papilionaceous | h | D | p | a | Pi | 55 | 50.6 | 11 | 0.15 |
| Phlox drummondii | Phlox drummondii | Polemoniaceae | A | S | herb | C3 | tube | h | D | p | p | Pu | 21 | 15 | 1.56 | 0.225 |
| Pilosocereus chrysacanthus | Pilosocereus chrysacanthus | Cactaceae | A | P | herb | CAM | tube | h | N | p | p | Pi | 36.41 | 93.94 | 75.17 | 4 |
| Platanthera hologlottis | Platanthera hologlottis | Orchidaceae | Z | P | herb | C3 | papilionaceous | h | B | p | p | W | 11 | 15 | 2 | 0.6 |
| Putoria calabrica | Plocama calabrica | Rubiaceae | A | P | shrub | C3 | tube | h | B | p | a | Pi | 6.29 | 14.09 | 28.33 | 0.11 |
| Polaskia chichipe | Polaskia chichipe | Cactaceae | A | P | herb | CAM | tube | h | D | p | p | G | 35 | 30 | 14 | 3 |
| Prunus persica | Prunus persica | Rosaceae | A | P | tree | C3 | open | h | B | p | p | Pi | 27.5 | 15 | 12 | 5 |
| Tillandsia macropetala | Pseudalcantarea macropetala | Bromeliaceae | A | P | herb | C3 | tube | h | N | p | p | G | 107 | 30 | 104 | 1.525 |
| Psittacanthus robustus | Psittacanthus robustus | Loranthaceae | A | P | vine | C3 | tube | h | D | p | a | Y | 5 | 110 | 100 | 7 |
| Pterocereus gaumeri | Pterocereus gaumeri | Cactaceae | A | P | herb | CAM | tube | h | N | p | p | Y | 51 | 50 | 40 | 8 |
| Randia itatiaiae | Randia itatiaiae | Rubiaceae | A | P | shrub | C3 | tube | m | N | p | p | W | 20.8 | 16.6 | 13 | 6.5 |
| Rubus chamaemorus | Rubus chamaemorus | Rosaceae | A | P | herb | C3 | open | d | D | a | a | W | 19 | 2.52 | 4.39 | 0.175 |
| Salix caprea | Salix caprea | Salicaceae | A | P | shrub | C3 | brush | d | N | p | p | G | 13.9 | 30.3 | 0.2 | 9 |
| Hylocereus costaricensis | Selenicereus costaricensis | Cactaceae | A | P | herb | CAM | tube | h | N | p | p | W | 250 | 260 | 200 | 4 |
| Hylocereus polyrhizus | Selenicereus monacanthus | Cactaceae | A | P | herb | CAM | tube | h | N | p | p | W | 70 | 135 | 200 | 4 |
| Hylocereus undatus | Selenicereus undatus | Cactaceae | A | P | herb | CAM | tube | h | N | p | p | W | 136 | 345 | 200 | 4 |
| Silene caroliniana | Silene caroliniana | Caryophyllaceae | A | P | herb | C3 | tube | h | D | p | a | Pi | 1.9 | 21.2 | 24.1 | 0.15 |
| Silene ciliata | Silene ciliata | Caryophyllaceae | A | P | herb | C3 | tube | h | N | p | a | Pi | 4 | 12.5 | 13.5 | 0.02 |
| Silene latifolia | Silene latifolia | Caryophyllaceae | A | P | herb | C3 | tube | d | N | p | p | W | 24 | 27 | 17 | 0.45 |
| Silene alba | Silene latifolia subsp. alba | Caryophyllaceae | A | P | herb | C3 | tube | d | N | p | p | W | 9 | 22.3 | 28 | 0.65 |
| Silene lemmonii | Silene lemmonii | Caryophyllaceae | A | P | herb | C3 | tube | h | N | a | a | W | 3 | 8 | 16 | 0.55 |
| Silene nutans | Silene nutans | Caryophyllaceae | A | P | herb | C3 | tube | d | N | p | p | W | 3 | 10.9 | 14.5 | 0.42 |
| Silene sennenii | Silene sennenii | Caryophyllaceae | A | P | herb | C3 | tube | h | N | a | p | Pi | 5 | 11 | 10 | 0.55 |
| Silene stellata | Silene stellata | Caryophyllaceae | A | P | herb | C3 | tube | h | N | a | p | W | 8 | 9.8 | 20.1 | 1.2 |
| Silene virginica | Silene virginica | Caryophyllaceae | A | P | herb | C3 | tube | h | D | p | a | R | 3.6 | 24.1 | 31.3 | 0.3 |
| Viscaria vulgaris | Silene viscaria | Caryophyllaceae | A | P | herb | C3 | tube | h | B | p | p | Pi | 20 | 12 | 15 | 0.375 |
| Silene vulgaris | Silene vulgaris | Caryophyllaceae | A | P | herb | C3 | tube | h | B | p | p | Pi | 20 | 12 | 15 | 0.375 |
| Echinopsis schickendantzii | Soehrensia schickendantzii | Cactaceae | A | P | herb | CAM | tube | h | N | a | p | W | 175 | 180 | 115 | 2 |
| Echinopsis thelegona | Soehrensia thelegona | Cactaceae | A | P | herb | CAM | tube | h | N | p | p | W | 142.1 | 182.5 | 133 | 2.5 |
| Sonneratia caseolaris | Sonneratia caseolaris | Lythraceae | A | P | tree | C3 | open | h | N | p | p | W | 55 | 57.5 | 82 | 15 |
| Stachyurus praecox | Stachyurus praecox | Stachyuraceae | A | P | shrub | C3 | campanulate | d | B | p | p | Y | 8 | 5 | 5 | 2.9 |
| Stenocereus queretaroensis | Stenocereus queretaroensis | Cactaceae | A | P | herb | CAM | tube | h | N | p | p | W | 48 | 88.77 | 70.03 | 10 |
| Stenocereus quevedonis | Stenocereus quevedonis | Cactaceae | A | P | herb | CAM | tube | h | N | p | p | W | 43 | 75 | 40 | 6 |
| Stenocereus stellatus | Stenocereus stellatus | Cactaceae | A | P | herb | CAM | tube | h | N | p | p | Pi | 35 | 67.5 | 37.5 | 4 |
| Stenocereus thurberi | Stenocereus thurberi | Cactaceae | A | P | herb | CAM | tube | h | N | p | p | W | 18.7 | 79.2 | 58 | 3.4 |
| Syzygium laetum | Syzygium laetum | Myrtaceae | A | P | tree | C3 | brush | h | B | p | p | R | 60.3 | 56.4 | 44.9 | 10 |
| Syzygium mundagam | Syzygium mundagam | Myrtaceae | A | P | tree | C3 | brush | h | B | p | p | W | 43.8 | 37.1 | 24.8 | 15 |
| Syzygium sayeri | Syzygium sayeri | Myrtaceae | A | P | tree | C3 | brush | h | D | p | a | W | 4.47 | 29.48 | 16.19 | 22.5 |
| Tilia americana | Tilia americana | Malvaceae | A | P | tree | C3 | open | h | N | p | p | Y | 12 | 4 | 4 | 27.5 |
| Tilia cordata | Tilia cordata | Malvaceae | A | P | tree | C3 | open | h | N | p | p | Y | 7 | 4 | 4 | 30 |
| Tilia platyphyllos | Tilia platyphyllos | Malvaceae | A | P | tree | C3 | open | h | N | p | p | Y | 14.5 | 4 | 4 | 30 |
| Tillandsia heterophylla | Tillandsia heterophylla | Bromeliaceae | A | P | herb | C3 | tube | h | N | p | p | W | 14 | 10 | 70 | 1.5 |
| Tournefortia rufo-sericea | Tournefortia rufo-sericea | Boraginaceae | A | P | shrub | C3 | tube | h | B | p | p | W | 4 | 5 | 2 | 2.5 |
| Trichosanthes anguina | Trichosanthes cucumerina | Cucurbitaceae | A | S | herb | C3 | tube | m | N | p | p | W | 37.5 | 27.5 | 27.5 | 2.5 |
| Trichosanthes kirilowii | Trichosanthes kirilowii | Cucurbitaceae | A | S | herb | C3 | tube | m | N | p | p | W | 25 | 12 | 12 | 6 |
| Trifolium pratense | Trifolium pratense | Fabaceae | Z | S | herb | C3 | papilionaceous | h | B | p | p | Pi | 3.5 | 16 | 16 | 0.5 |
| Vaccinium angustifolium | Vaccinium angustifolium | Ericaceae | A | P | shrub | C3 | campanulate | h | B | p | p | W | 5 | 5 | 4 | 0.325 |
| Vitis rotundifolia | Vitis rotundifolia | Vitaceae | A | P | vine | C3 | open | d | B | p | p | G | 3.5 | 3.5 | 1 | 21 |
| Yucca aloifolia | Yucca aloifolia | Asparagaceae | A | P | herb | CAM | campanulate | h | N | a | p | W | 55 | 35 | 17.5 | 2 |
| Yucca elata | Yucca elata | Asparagaceae | A | P | herb | CAM | campanulate | h | N | a | p | W | 59.5 | 44.5 | 10 | 5 |
| Yucca filamentosa | Yucca filamentosa | Asparagaceae | A | P | herb | C3 | campanulate | h | N | a | p | W | 35 | 35 | 25 | 2.75 |
| Fragaria x ananassa | Fragaria ananassa | Rosaceae | A | P | herb | C3 | open | h | B | p | p | W | 31.2 | 31.2 | 1 | 0.2 |
| Fontainea picrosperma | Fontainea picrosperma | Euphorbiaceae | A | P | tree | C3 | open | d | B | a | p | W | 19.5 | 6.5 | 4 | 25 |
| Rubus fruticosus | Rubus fruticosus | Rosaceae | A | P | shrub | C3 | open | h | B | p | p | W | 22.5 | 7.5 | 4 | 2.5 |
| Clarkia concinna | Clarkia concinna | Onagraceae | Z | S | herb | C3 | open | h | B | p | p | Pi | 56.9 | 17.5 | 14.6 | 0.22 |
| Clarkia breweri | Clarkia breweri | Onagraceae | Z | S | herb | C3 | open | h | B | p | p | Pi | 48.4 | 12.5 | 23.1 | 0.175 |
| Habenaria dentata | Habenaria dentata | Orchidaceae | Z | P | herb | C3 | papilionaceous | h | D | p | a | W | 6.97 | 44.9 | 2 | 0.575 |
| Lonicera japonica | Lonicera japonica | Caprifoliaceae | Z | P | vine | C3 | tube | h | N | p | a | W | 15 | 40 | 40 | 10 |
| Banksia spinulosa | Banksia spinulosa | Proteaceae | A | P | shrub | C3 | brush | h | B | p | p | Y | 65 | 105 | 22.5 | 2 |

**Table S2.** Summary statistics for each univariate meta-regression day vs. night pollination in relation to environmental and plant trait variables. *Q*_M_: Omnibus (Wald-type) test statistic for each moderator, along with its degree of freedom and adjusted p-value. R^2^_M_ and R^2^_C_: Marginal and conditional R^2^. QE: test statistic for residual heterogeneity, along with degrees of freedom and p-value. Variables are in descending order of R^2^_M_.

| Variable | *Q*_M_ | df | p-value | R^2^_M_ | R^2^_C_ | *Q_E_* | df | p-value |
| --- | --- | --- | --- | --- | --- | --- | --- | --- |
| Elevation^2^ | 29.948 | 3 | < 0.001 | 0.139 | 0.766 | 6065.013 | 396 | < 0.001 |
| Bloom period | 23.258 | 3 | < 0.001 | 0.118 | 0.746 | 5672.969 | 396 | < 0.001 |
| Flower colour | 30.511 | 7 | 0.0014 | 0.117 | 0.736 | 5254.171 | 392 | < 0.001 |
| Odour | 25 | 2 | < 0.001 | 0.107 | 0.743 | 5989.854 | 397 | < 0.001 |
| Pollination dependency^2^ | 8.128 | 3 | 0.1736 | 0.053 | 0.822 | 2357.905 | 171 | < 0.001 |
| Pollination dependency | 7.319 | 2 | 0.1736 | 0.053 | 0.822 | 2359.062 | 172 | < 0.001 |
| DTR^2^ | 11.3 | 3 | 0.1224 | 0.047 | 0.734 | 6161.288 | 396 | < 0.001 |
| PS pathway | 9.342 | 2 | 0.1224 | 0.046 | 0.741 | 5806.9 | 397 | < 0.001 |
| Life form | 10.563 | 4 | 0.1736 | 0.045 | 0.74 | 6151.025 | 395 | < 0.001 |
| Flower shape | 10.097 | 5 | 0.261 | 0.045 | 0.744 | 5905.233 | 394 | < 0.001 |
| Breeding system | 8.658 | 3 | 0.1736 | 0.043 | 0.746 | 6144.409 | 396 | < 0.001 |
| DTR | 8.031 | 2 | 0.1736 | 0.037 | 0.732 | 6171.469 | 397 | < 0.001 |
| Daylength | 7.534 | 2 | 0.1736 | 0.037 | 0.742 | 6167.524 | 397 | < 0.001 |
| Daylength^2^ | 7.785 | 3 | 0.1919 | 0.035 | 0.741 | 6165.456 | 396 | < 0.001 |
| Lifespan | 6.805 | 2 | 0.1736 | 0.034 | 0.742 | 6198.217 | 397 | < 0.001 |
| Flower symmetry | 7.569 | 2 | 0.1736 | 0.033 | 0.741 | 6021.005 | 397 | < 0.001 |
| Plant height | 6.68 | 2 | 0.1736 | 0.03 | 0.741 | 6198.722 | 397 | < 0.001 |
| Nectar | 6.605 | 2 | 0.1736 | 0.03 | 0.739 | 6004.506 | 397 | < 0.001 |
| Elevation | 6.376 | 2 | 0.1736 | 0.029 | 0.738 | 6172.972 | 397 | < 0.001 |
| Style length | 6.351 | 2 | 0.1736 | 0.029 | 0.741 | 6162.255 | 397 | < 0.001 |

**Identification and screening of studies for meta-analysis**

Records identified from:

9 WoS Databases (n = 1893)

Buxton et al. 2022 (n = 173)

MacGregor & Scott-Brown 2020 (n = 133)

Duplicate records removed *before screening*:

(n =249)

**Identification**

Records screened:

(n = 1950)

Records excluded:

(n = 1675)

Studies sought for retrieval:

(n = 275)

Studies not retrieved

(n = 0)

**Screening**

Studies excluded from extraction:

(n = 139)

Studies assessed for eligibility

(n = 275)

Studies included in review

(n = 136)

Studies excluded from analyses:

Incompatible traits

(n = 1)

**Included**

**Figure A1.1.** PRISMA flow diagram for the systematic review detailing database searches, screening procedures and the number of studies with data included in the meta-analysis.


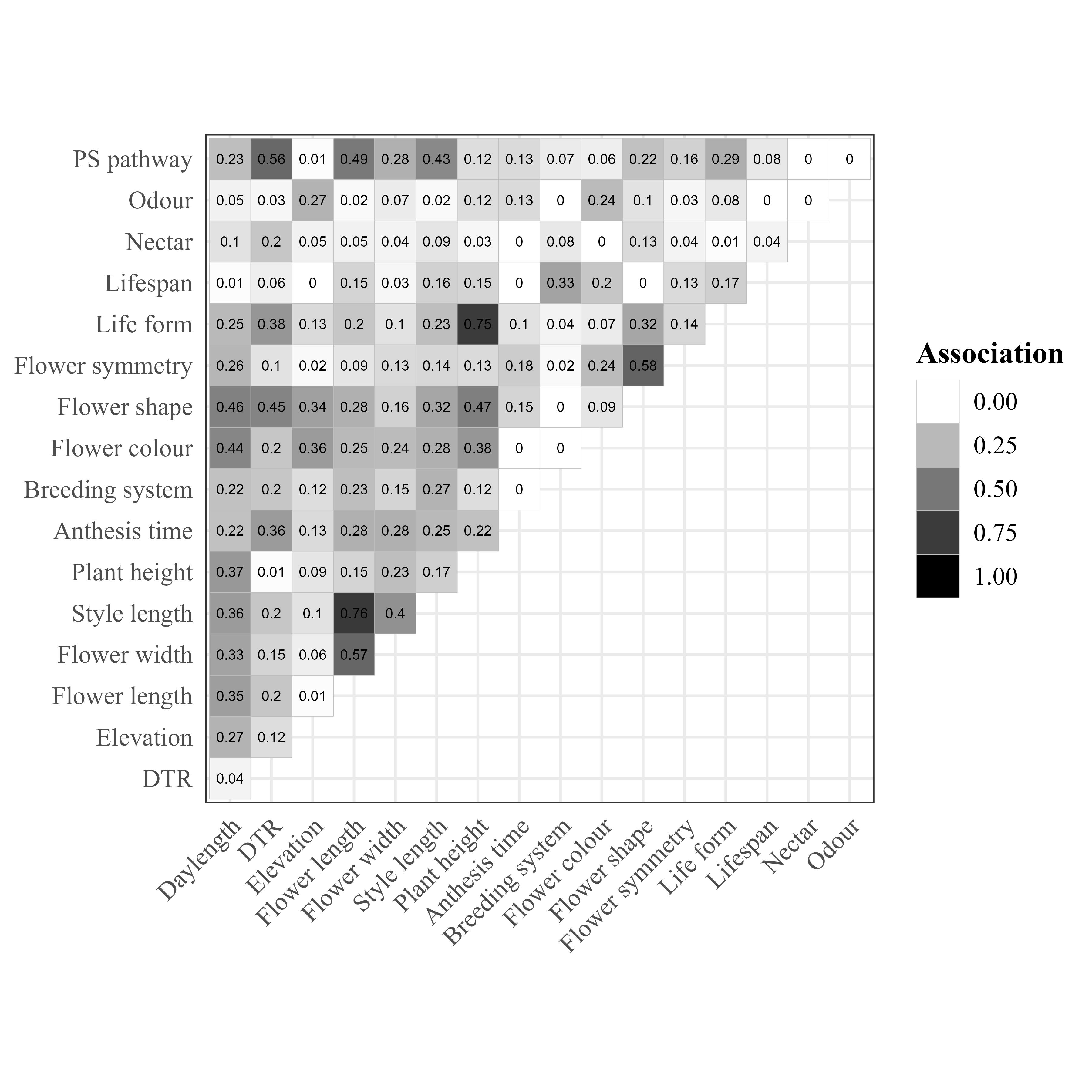


**Figure A1.2.** Pairwise associations between plant species traits and study environmental variables. The value of association is either Cramer’s V for pairs of nominal variables, Pearson’s correlation co-efficient for pairs of continuous variables, or R^2^ between nominal ~ continuous pairs. Rows are ordered with categorical traits first, then continuous traits and lastly environmental variables.

**
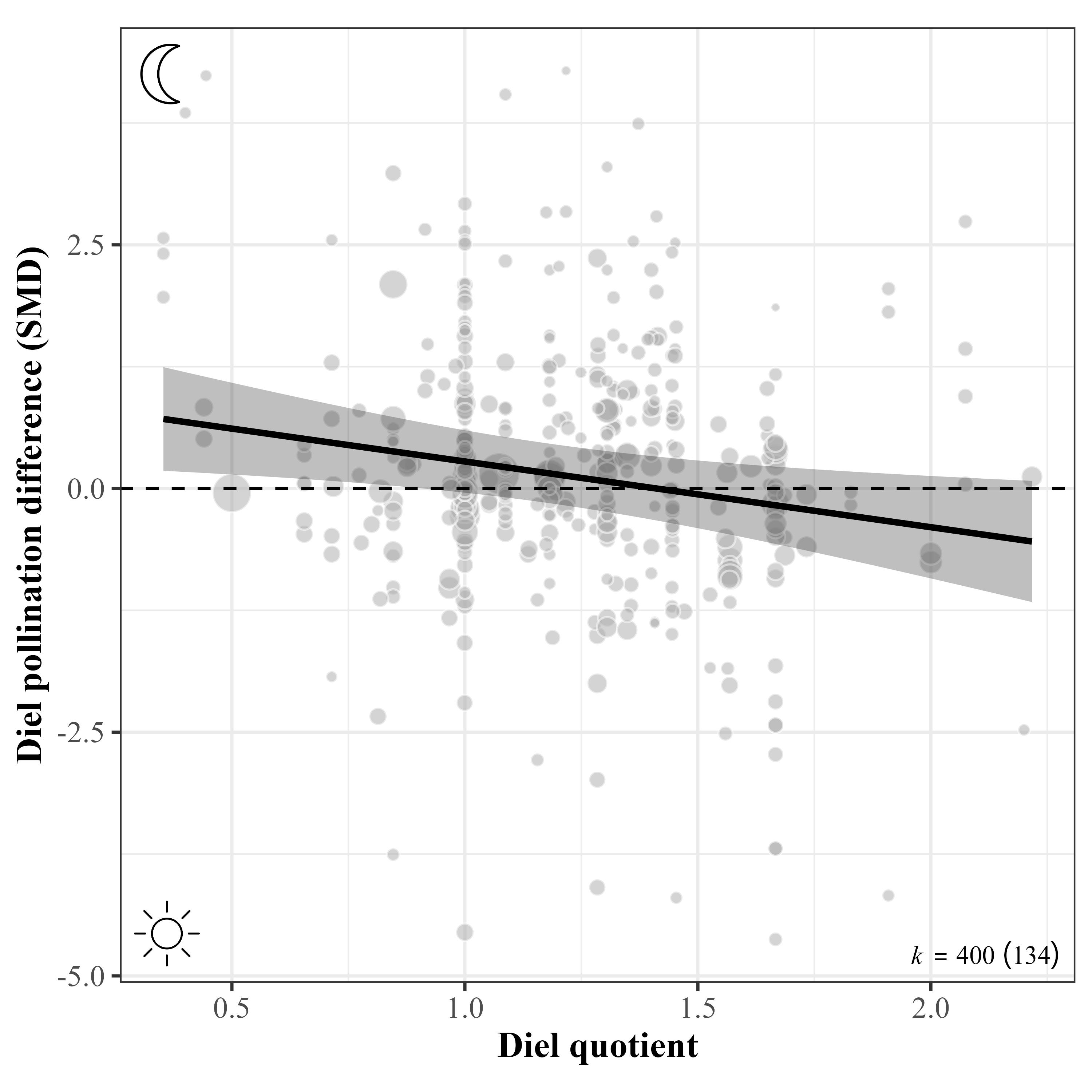
**

**Figure A1.3.** Diel quotient in relation to the diel pollination difference (standardised mean difference, SMD) between day and night pollination. Diel quotient is calculated as the ratio of day pollination hours to night pollination hours, thus the negative relationship shown indicates an increase in day pollination effect with increasing day pollination exposure time. Solid line and shaded ribbon indicate predicted line of best fit and 95% confidence intervals. Background points indicate individual effect sizes, in which size is proportional to the inverse of the standard error of the effect size. *k =* the number of effect sizes along with the number of studies in parentheses.

**
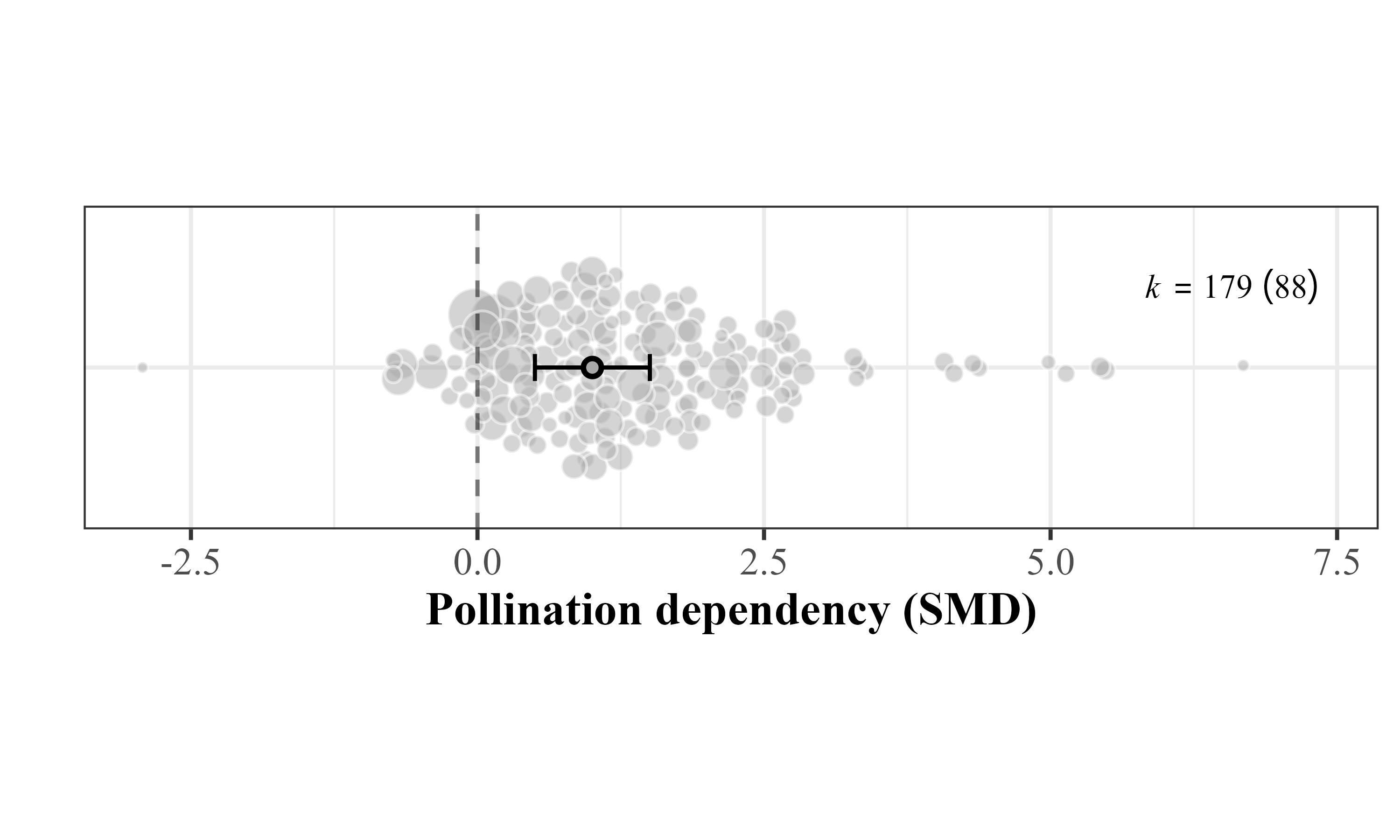
**

**Figure A1.4.** Species pollination dependency, measured as the standardised mean difference (SMD) between closed and open pollination treatments. Primary dot and error bar indicate the predicted mean estimates ± standard error. Background points indicate individual effect sizes, in which size is proportional to the inverse of the standard error of the effect size. *k =* the number of effect sizes along with the number of studies in parentheses.


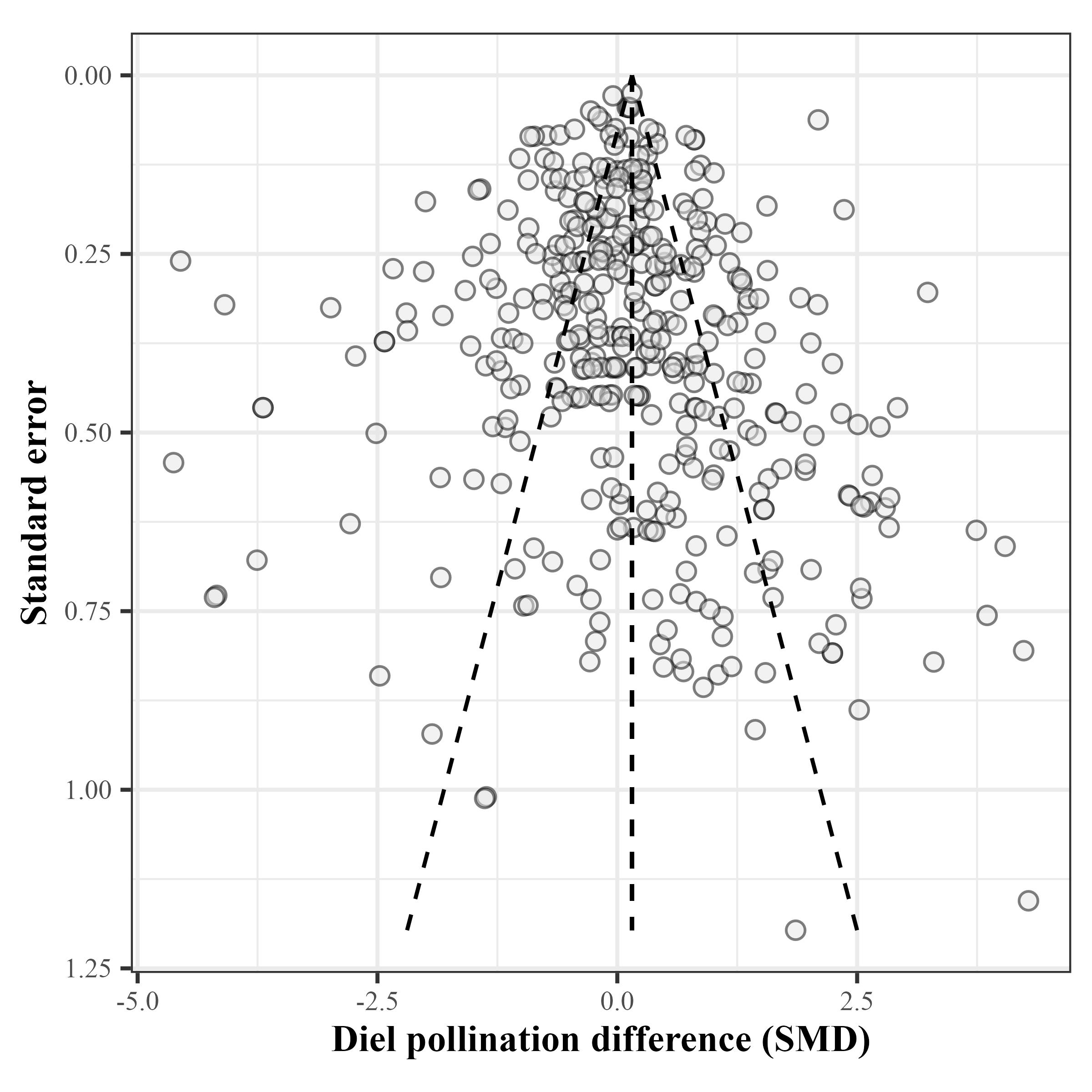


**Figure A1.5.** Funnel plots showing the relationship between diel pollination difference (standardized mean difference, SMD) between day and night pollination and standard error. Dashed lines indicate the predicted mean effect size and 95% pseudo-confidence intervals (i.e., 1.96 ± SE). Background points are individual effect sizes.

**APPENDIX 2: Supplemental results for comparisons between day and night pollination and open pollination**

**Table A2.1.** Total heterogeneity (I^2^) in effect sizes and partial heterogeneity attributable to each random effect in the meta-analytic model for each diel ~ open pollination comparison.

| Comparison | I^2^_total_ | I^2^_effect_ | I^2^_measure_ | I^2^_phylo_ | I^2^_species_ | I^2^_study_ |
| --- | --- | --- | --- | --- | --- | --- |
| Day vs. open | 96 | 34 | 26 | 6 | 15 | 15 |
| Night vs. open | 96 | 27 | 13 | 4 | 48 | 4 |

Phylogenetic meta-analysis revealed that there were no overall significant differences between either day or night pollination and open pollination (**Figure A2.1 & Figure A2.2**). Total heterogeneity for each comparative dataset was high (96 %, **Table A2.1),** with varying amounts attributable to each random effect, depending on the contrast. Furthermore, we found evidence for phylogenetic signal in differences between day and open pollination (λ = 0.330, p < 0.001), but not night and open pollination (λ = 0.047, p = 0.672).


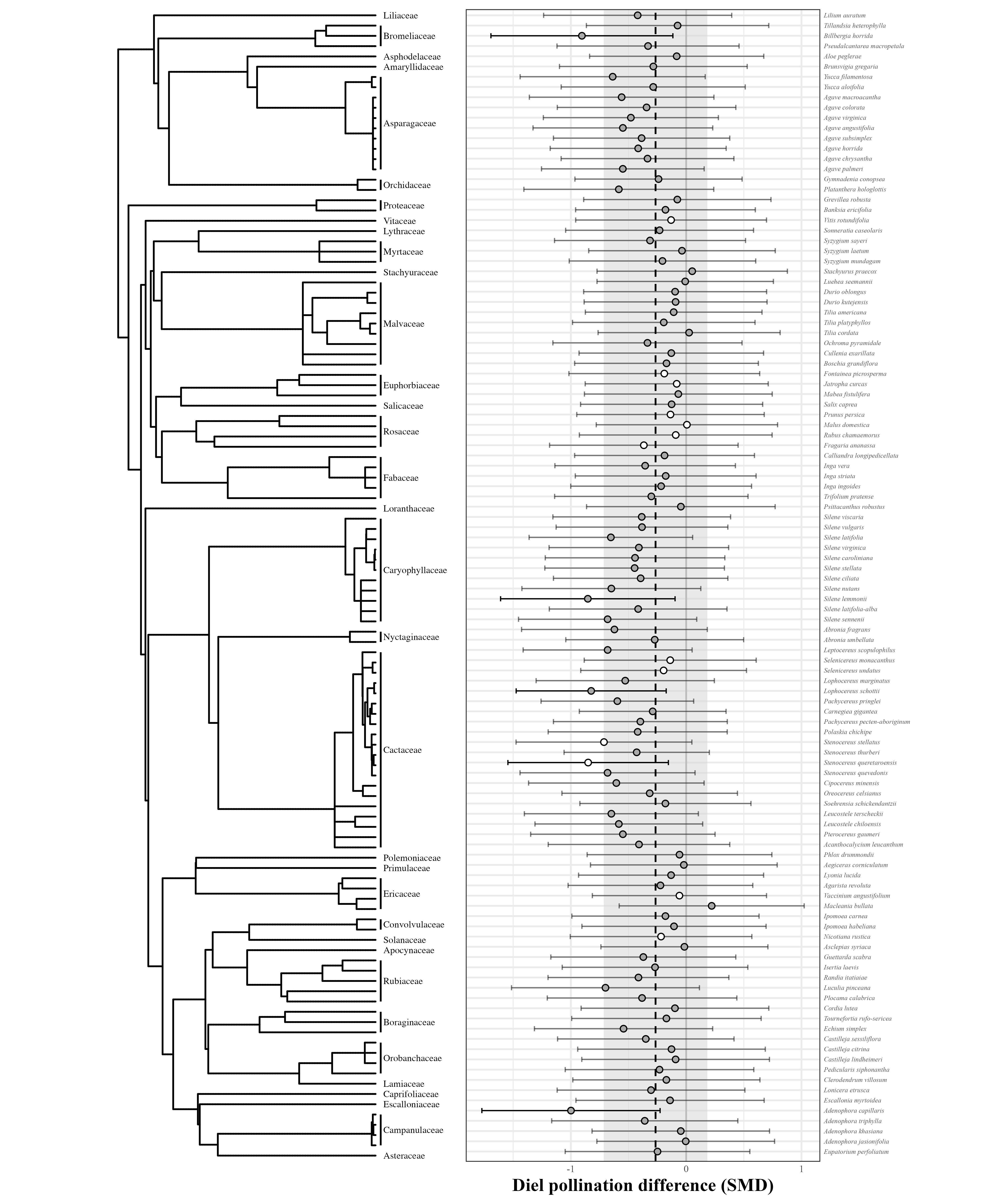


**Figure A2.1**. Phylogeny of plant species included in meta-analysis (left), and species-level predictions of diel pollination difference between day and open pollination (right). Primary dots and error bars indicate marginal species-level mean estimates ± standard error. Filled circles indicate wild plant species, and open circles are crop species. Vertical dashed line and shaded rectangle indicate meta-analytical mean estimate and 95% confidence interval.


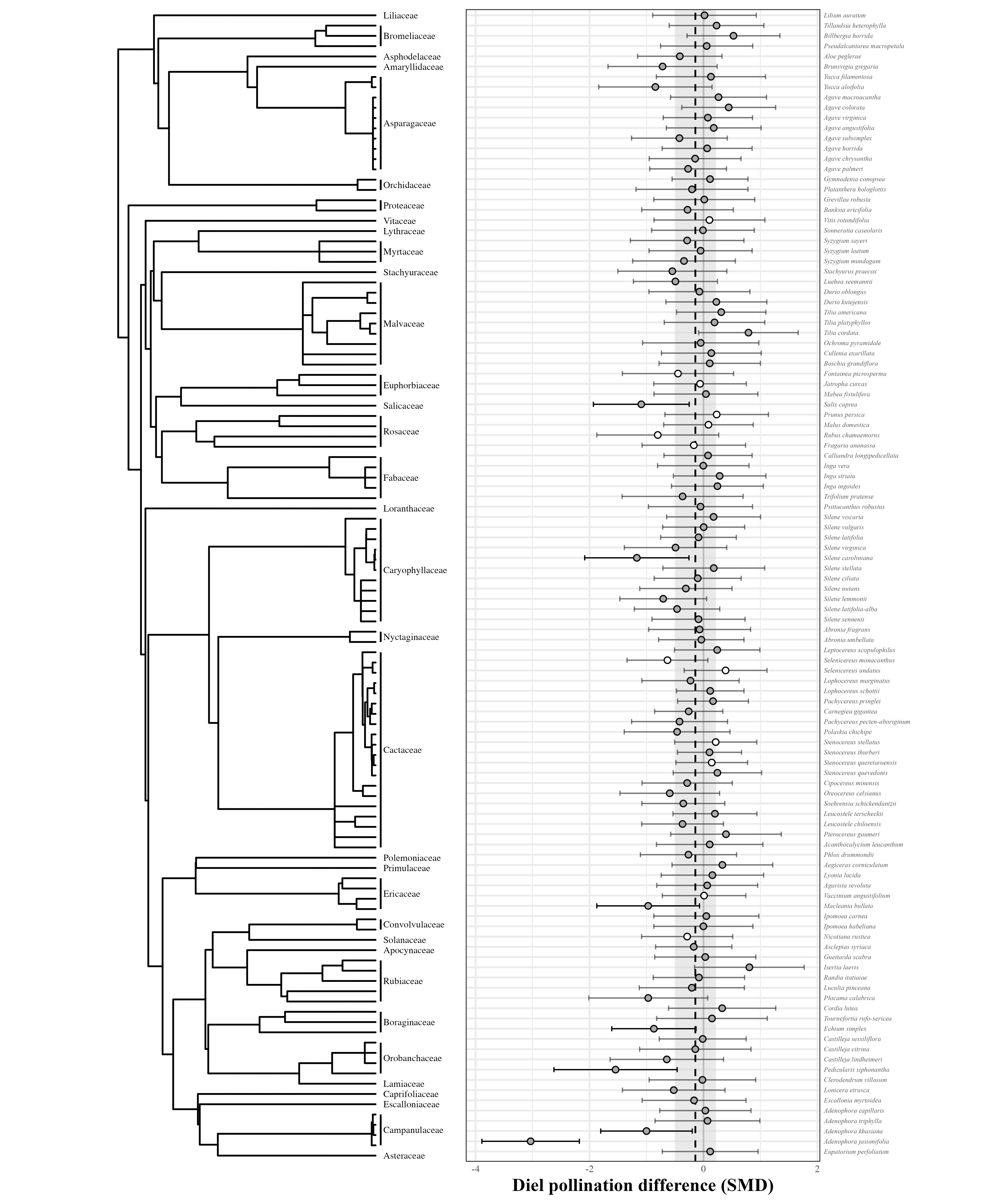


**Figure A2.2**. Phylogeny of plant species included in meta-analysis (left), and species-level predictions of diel pollination difference between night and open pollination (right). Primary dots and error bars indicate marginal species-level mean estimates ± standard error. Filled circles indicate wild plant species, and open circles are crop species. Vertical dashed line and shaded rectangle indicate meta-analytical mean estimate and 95% confidence interval.

At the pollination measurement level (**Figure A2.3)**, fruit set and seed set resulting from day pollination were significantly lower than open pollination (fruit set: -0.451 [-0.652, -0.251], seed set: -0.502 [-0.723, -0.281]) and seed mass was significantly higher than open pollination (0.573 [0.109, 1.036]). Furthermore, fruit set resulting from night pollination was significantly less than open pollination (-0.41 [-0.622, -0.198]), whereas seed mass was significantly higher than open pollination (0.471 [0.031, 0.911]).


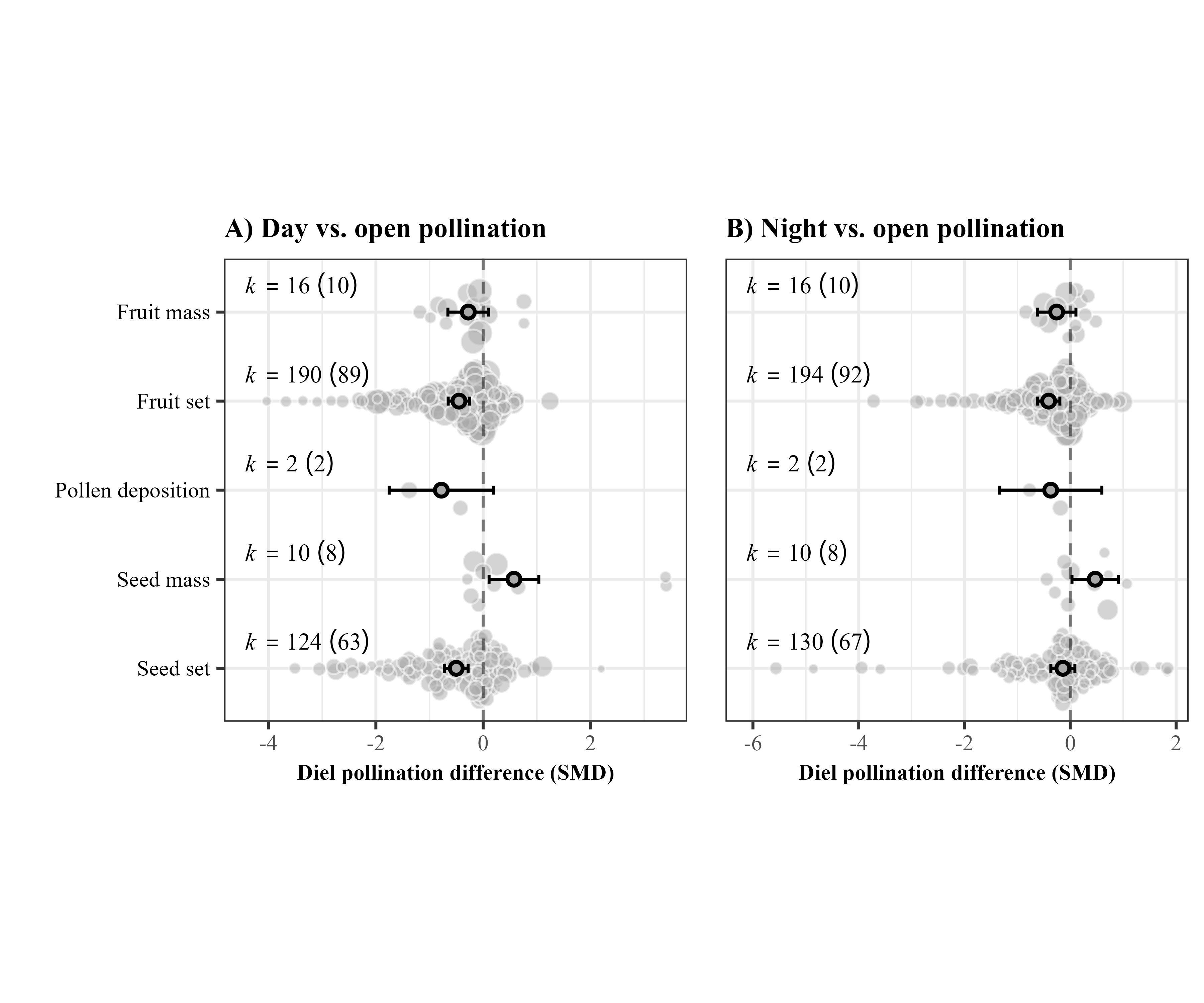


**Figure A2.3**. Diel pollination differences (Standardized mean differences, SMD) between day vs. open pollination (A) and night vs. open pollination (B) for each pollination outcome measure. Primary dots and error bars indicate marginal mean estimates and 95% confidence intervals. Background points indicate individual effect sizes, in which size is proportional to the inverse of the standard error of the effect size. *k =* the number of effect sizes along with the number of studies in parentheses.

*Diel pollination differences in relation to environmental variables and plant traits*

**Table A2.2.** Summary statistics for each univariate meta-regression of environmental and plant trait variables in each diel ~ open pollination comparison. Q_M_: Cochrane’s Q test statistic for each moderator, along with its degree of freedom and adjusted p-value. R^2^_M_ and R^2^_C_: Marginal and conditional R^2^ . Q_E_: test statistic for residual heterogeneity, along with degrees of freedom and p-value.

| Comparison | Variable | *Q*_M_ | df | p-value | R^2^_M_ | R^2^_C_ | *Q*_E_ | df | p-value |
| --- | --- | --- | --- | --- | --- | --- | --- | --- | --- |
| Day vs. open | Flower colour | 22.821 | 7 | 0.0576 | 0.08 | 0.682 | 2679.072 | 334 | < 0.001 |
|  | Life form | 18.905 | 4 | 0.0516 | 0.067 | 0.662 | 2993.795 | 337 | < 0.001 |
|  | Flower shape | 14.274 | 5 | 0.2196 | 0.062 | 0.648 | 2785.358 | 336 | < 0.001 |
|  | Bloom period | 11.381 | 3 | 0.2063 | 0.057 | 0.657 | 2824.676 | 338 | < 0.001 |
|  | Elevation^2^ | 8.271 | 3 | 0.427 | 0.036 | 0.659 | 3193.427 | 338 | < 0.001 |
|  | PS pathway | 6.505 | 2 | 0.427 | 0.04 | 0.65 | 2718.422 | 339 | < 0.001 |
|  | Odour | 5.394 | 2 | 0.6058 | 0.021 | 0.659 | 3195.591 | 339 | < 0.001 |
|  | Nectar | 4.837 | 2 | 0.6673 | 0.022 | 0.641 | 3023.292 | 339 | < 0.001 |
|  | Style length | 4.698 | 2 | 0.6673 | 0.02 | 0.658 | 3120.956 | 339 | < 0.001 |
|  | Daylength^2^ | 4.224 | 3 | 0.9369 | 0.015 | 0.659 | 3166.506 | 338 | < 0.001 |
|  | Lifespan | 4.178 | 2 | 0.7788 | 0.017 | 0.652 | 3195.726 | 339 | < 0.001 |
|  | Plant height | 3.741 | 2 | 0.872 | 0.015 | 0.654 | 3074.98 | 339 | < 0.001 |
|  | Breeding system | 3.706 | 3 | 1 | 0.015 | 0.656 | 3175.736 | 338 | < 0.001 |
|  | Daylength | 3.588 | 2 | 0.872 | 0.015 | 0.656 | 3176.826 | 339 | < 0.001 |
|  | DTR^2^ | 3.355 | 3 | 1 | 0.013 | 0.653 | 3116.481 | 338 | < 0.001 |
|  | Elevation | 3.149 | 2 | 0.9369 | 0.013 | 0.653 | 3196.129 | 339 | < 0.001 |
|  | DTR | 2.988 | 2 | 0.9369 | 0.012 | 0.651 | 3126.604 | 339 | < 0.001 |
|  | Flower symmetry | 2.96 | 2 | 0.9369 | 0.012 | 0.653 | 3131.166 | 339 | < 0.001 |
| Night vs. open | Odour | 35.594 | 2 | < 0.001 | 0.161 | 0.725 | 2440.994 | 349 | < 0.001 |
|  | Elevation^2^ | 31.807 | 3 | < 0.001 | 0.15 | 0.723 | 2498.361 | 348 | < 0.001 |
|  | Flower colour | 17.824 | 7 | 0.161 | 0.086 | 0.729 | 2486.395 | 344 | < 0.001 |
|  | Flower shape | 11.606 | 5 | 0.3649 | 0.066 | 0.735 | 2639.915 | 346 | < 0.001 |
|  | Life form | 10.034 | 4 | 0.3649 | 0.059 | 0.74 | 2529.484 | 347 | < 0.001 |
|  | Elevation | 9.882 | 2 | 0.1443 | 0.053 | 0.703 | 2695.329 | 349 | < 0.001 |
|  | Plant height | 9.383 | 2 | 0.1443 | 0.046 | 0.728 | 2587.794 | 349 | < 0.001 |
|  | Bloom period | 7.039 | 3 | 0.5556 | 0.044 | 0.737 | 2625.253 | 348 | < 0.001 |
|  | DTR^2^ | 6.434 | 3 | 0.6453 | 0.027 | 0.723 | 2663.848 | 348 | < 0.001 |
|  | Breeding system | 5.88 | 3 | 0.7275 | 0.035 | 0.728 | 2597.6 | 348 | < 0.001 |
|  | Flower symmetry | 4.124 | 2 | 0.7275 | 0.023 | 0.727 | 2686.586 | 349 | < 0.001 |
|  | Style length | 3.762 | 2 | 0.7645 | 0.022 | 0.725 | 2690.664 | 349 | < 0.001 |
|  | DTR | 3.691 | 2 | 0.7645 | 0.018 | 0.725 | 2671.757 | 349 | < 0.001 |
|  | Nectar | 2.683 | 2 | 1 | 0.016 | 0.728 | 2696.741 | 349 | < 0.001 |
|  | Daylength^2^ | 2.492 | 3 | 1 | 0.009 | 0.734 | 2697.231 | 348 | < 0.001 |
|  | PS pathway | 2.482 | 2 | 1 | 0.017 | 0.729 | 2694.991 | 349 | < 0.001 |
|  | Lifespan | 2.135 | 2 | 1 | 0.01 | 0.727 | 2687.992 | 349 | < 0.001 |
|  | Daylength | 1.803 | 2 | 1 | 0.008 | 0.728 | 2698.253 | 349 | < 0.001 |

Comparison of Cochrane’s Q test (**Table A2.2**), as well as the explained variance (**Figure A2.4**) by each environmental and trait variable (marginal R^2^) revealed that elevation and floral odour each explained a significant amount of variation for night vs. open pollination. In contrast, no tested variable explained a significant amount of variation for day vs. open pollination.


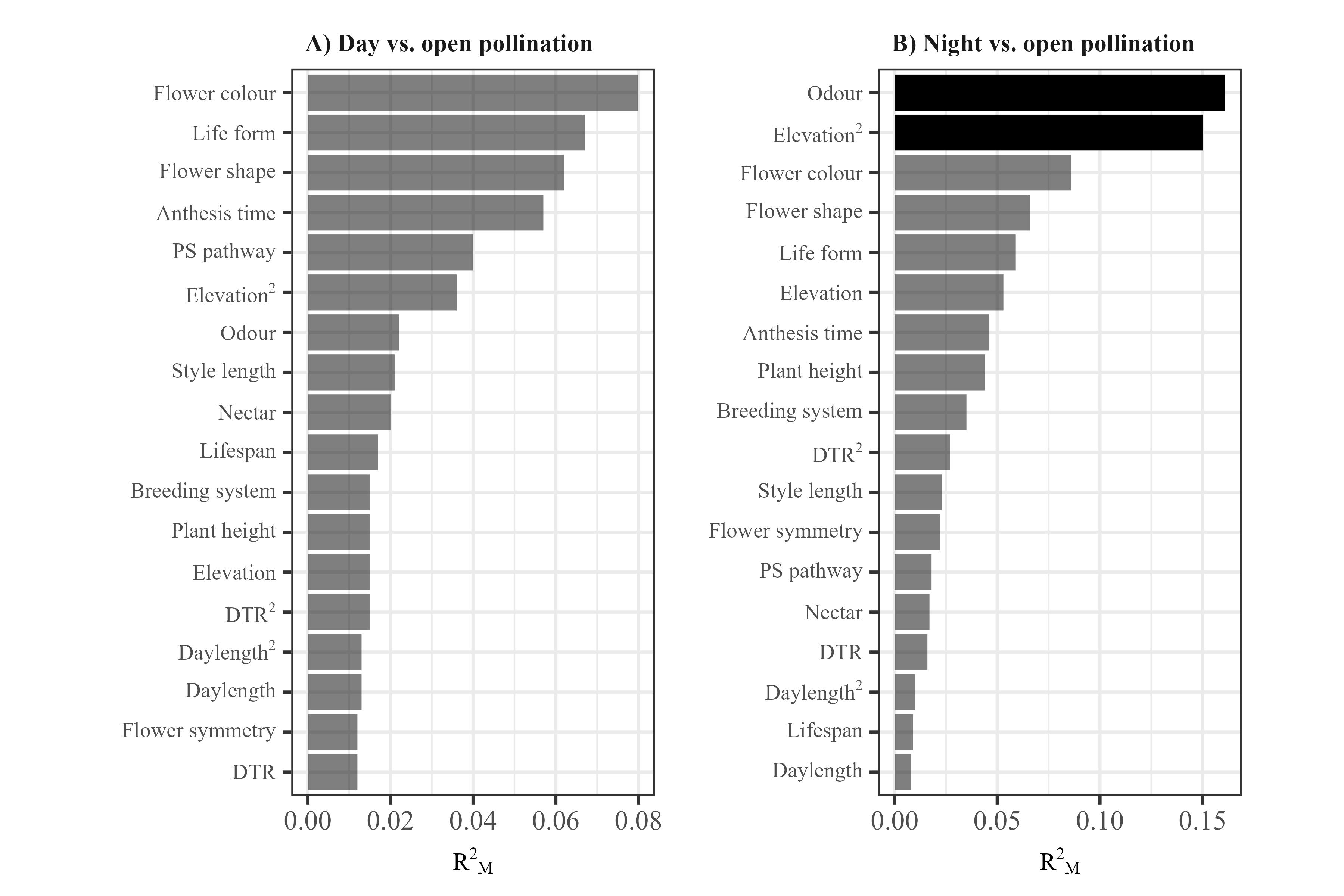


**Figure A2.4**. Coefficient of determination (marginal R^2^) for each environmental and trait variable in relation to the diel pollination differences between day vs. open pollination (A) and night vs. open pollination (B). Solid columns are those variables for which Cochrane’s Q test (*Q*_M_) was significant (p < 0.05), whereas transparent columns were non-significant (p > 0.05).

We found a significant negative quadratic relationship between the diel pollination difference between night and open pollination and elevation (linear term: z = 1.115, p = 0.265, quadratic term: z = -4.565, p < 0.001), such that the success of nocturnal pollination relative to open pollination declined at higher elevations (> 2000 m) (**Figure A2.5**).


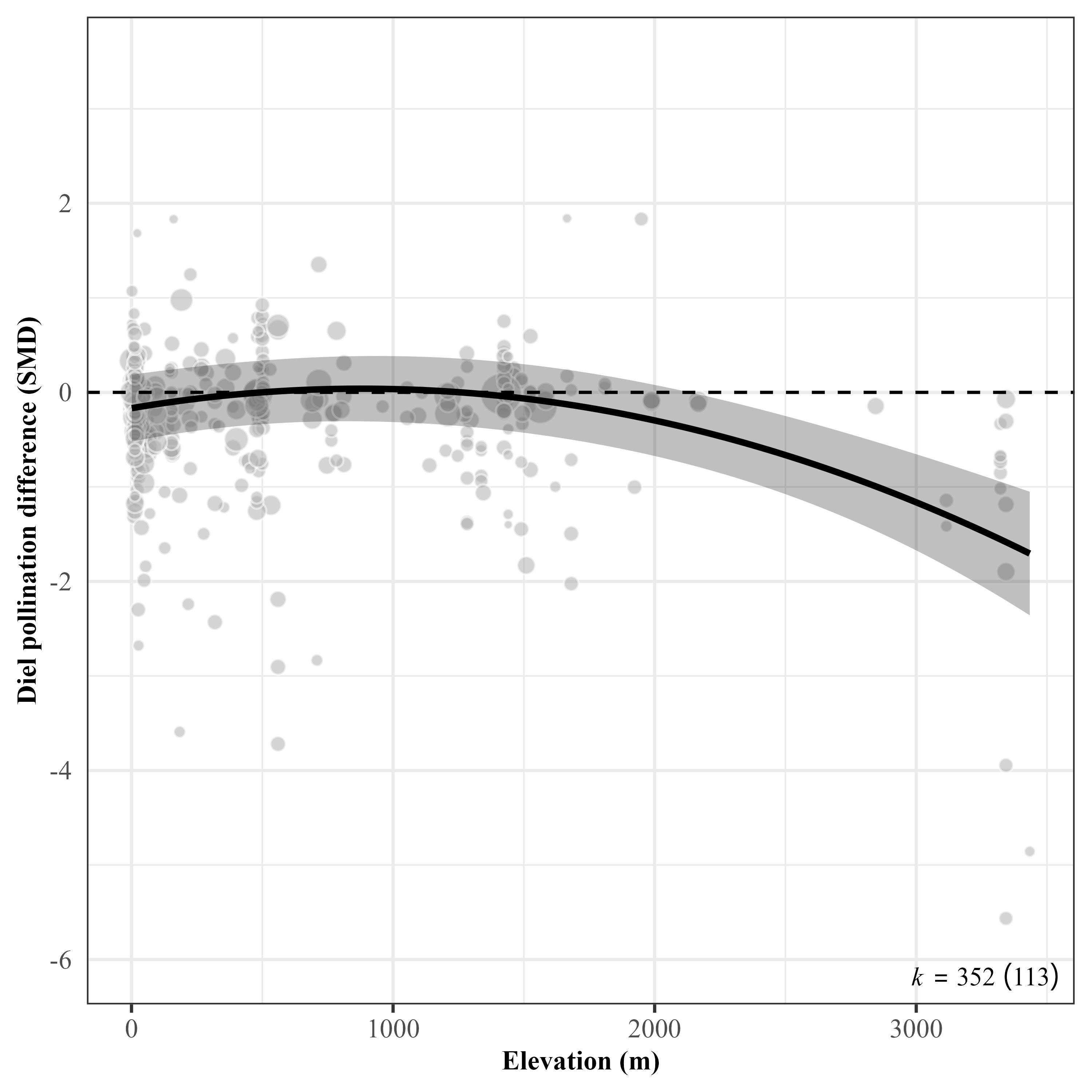


**Figure A2.5.** Diel pollination differences (Standardized mean differences, SMD) between day vs. open pollination (A) and night vs. open pollination (B) in relation to elevation (m). Solid line and shaded ribbon indicate predicted line of best fit and 95% confidence intervals. Background points indicate individual effect sizes, in which size is proportional to the inverse of the standard error of the effect size. *k =* the number of effect sizes along with the number of studies in parentheses.

Flowers without odour had significantly lower pollination success from nocturnal pollination, relative to open pollination (-1.094 [-1.559, -0.629]) (**Figure A2.6**).


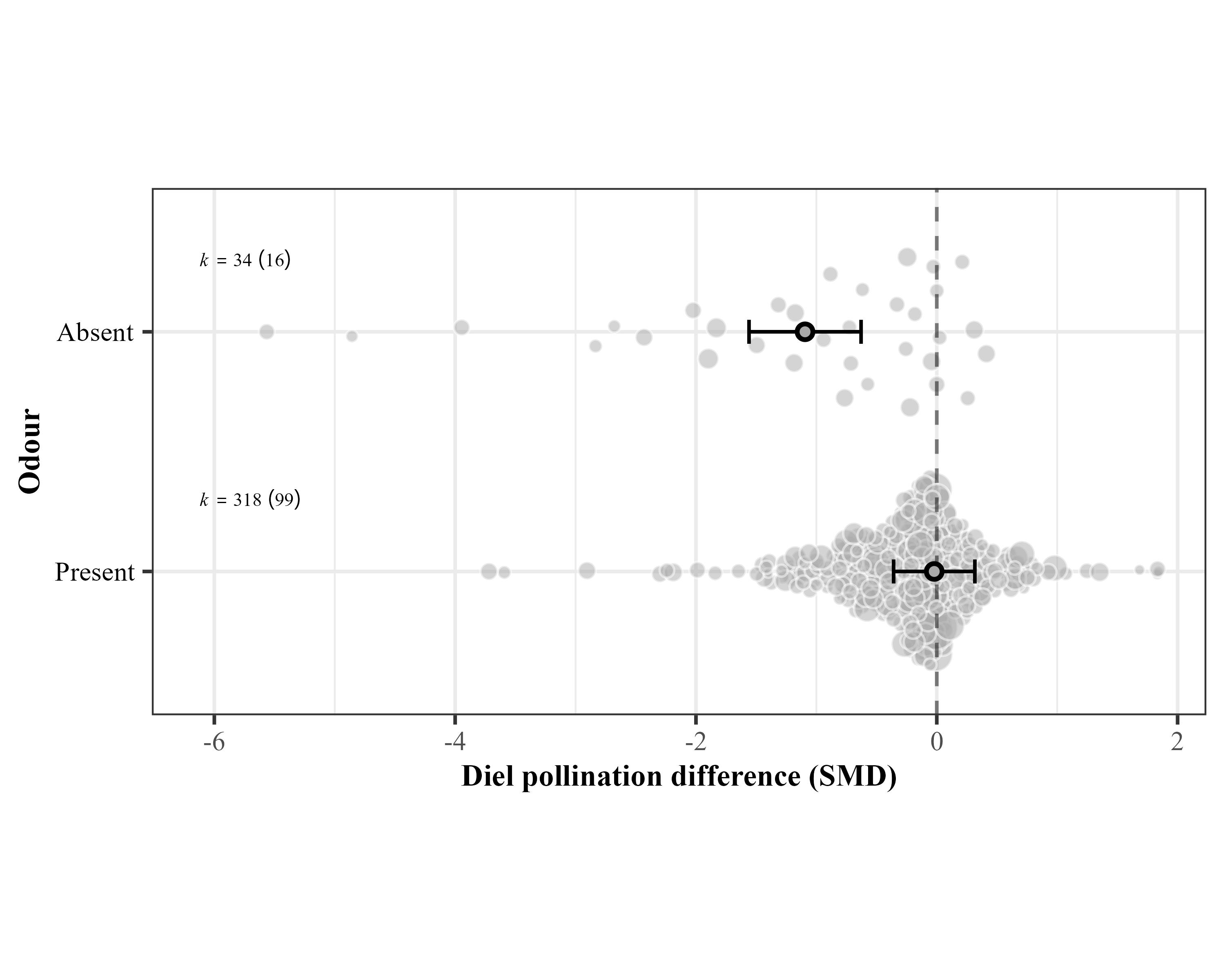


**Figure A2.6.** Diel pollination differences (Standardized mean difference, SMD) between night vs. open pollination in relation to flower odour. Primary dots and error bars indicate marginal mean estimates and 95% confidence intervals. Background points indicate individual effect sizes, in which size is proportional to the inverse of the standard error of the effect size. *k =* the number of effect sizes along with the number of studies in parentheses.

*Publication bias and sensitivity analysis*

Although there was some evidence for asymmetry in comparisons between diurnal or nocturnal pollination and open pollination (**Figure A2.7**), results from the Egger's tests suggested there was no evidence for asymmetry in comparisons with open pollination (day vs. open: z = 0.664, p = 0.507; night vs. open: z = 0.389, p = 0.697) and we found no evidence for a time-lag effect (year effect: day vs. open: z = -1.241, p = 0.215; night vs. open: z = -1.317, p = 0.188).

**
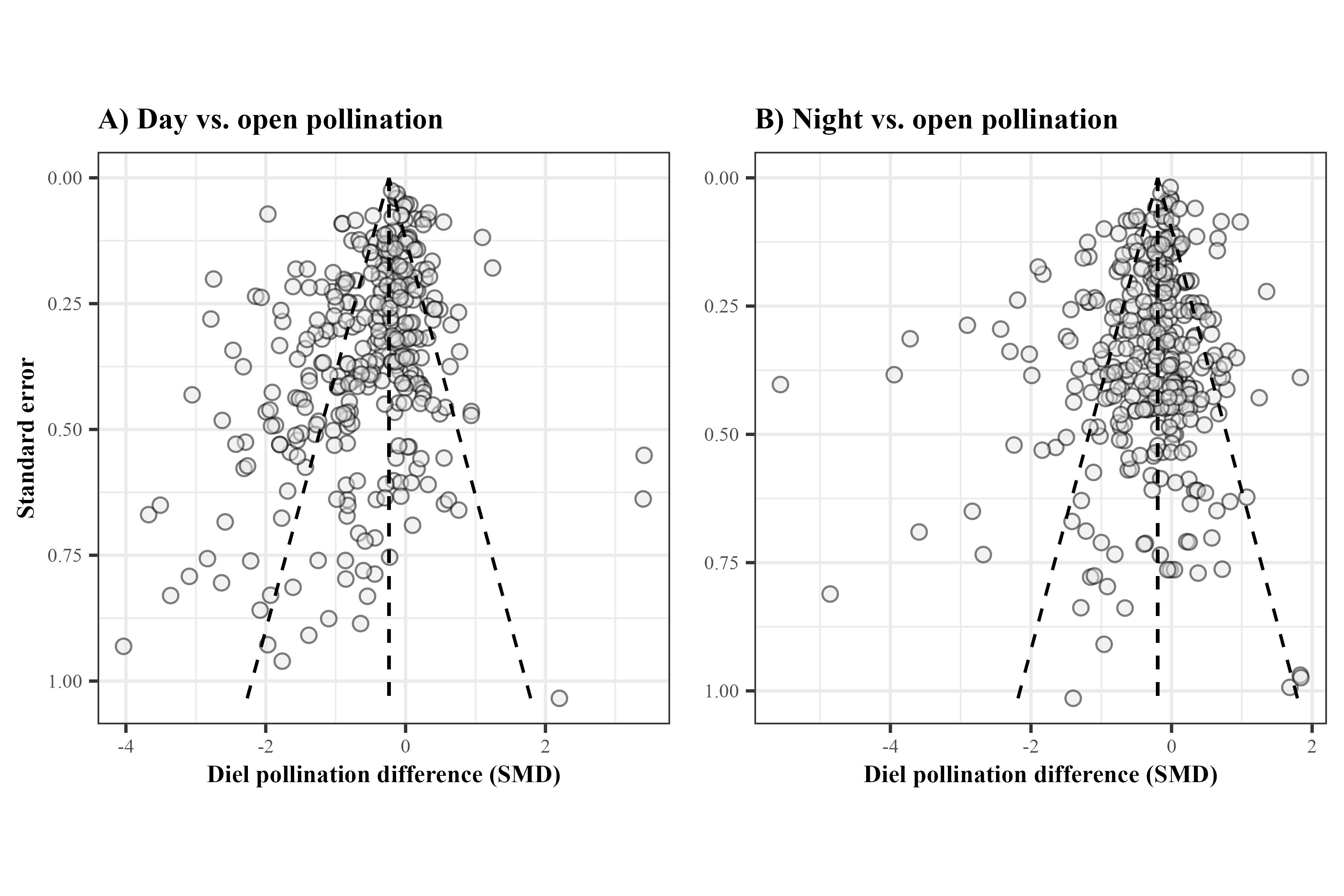
**

**Figure A2.7.** Funnel plots showing the relationship between the diel pollination differences (Standardized mean differences, SMD) between day vs. open pollination (A) and night vs. open pollination (B) and standard error. Dashed lines indicate the predicted mean effect size and 95% pseudo-confidence intervals (i.e., 1.96 ± SE). Background points are individual effect sizes.
